# Global burden of chikungunya virus infections and the potential benefit of vaccination campaigns

**DOI:** 10.1038/s41591-025-03703-w

**Published:** 2025-06-10

**Authors:** Gabriel Ribeiro dos Santos, Fariha Jawed, Christinah Mukandavire, Arminder Deol, Danny Scarponi, Leonard E. G. Mboera, Eric Seruyange, Mathieu J. P. Poirier, Samuel Bosomprah, Augustine O. Udeze, Koussay Dellagi, Nathanael Hozé, Jaffu Chilongola, Gheyath K. Nasrallah, Simon Cauchemez, Henrik Salje

**Affiliations:** 1 Department of Genetics, University of Cambridge, Cambridge, UK; 2 Department of Epidemiology of Microbial Diseases, Yale School of Public Health, New Haven, CT USA; 3 Coalition for Epidemic Preparedness Innovations, London, UK; 4 Sokoine University of Agriculture, Morogoro, Tanzania; 5 Rwanda Military Teaching Hospital and University of Rwanda, Kigali, Rwanda; 6 York University, Toronto, ON Canada; 7 Department of Biostatistics, School of Public Health, University of Ghana, Accra, Ghana; 8 University of Ilorin, Ilorin, Nigeria; 9 Pasteur Network, Paris, France; 10 Université Paris Cité, INSERM, IAME, Paris, France; 11 Institut Pasteur, Epidemiology and Modelling of Antimicrobials Evasion Research Unit, Paris, France; 12 Université Paris-Saclay, UVSQ, INSERM, CESP, Anti-infective Evasion and Pharmacoepidemiology Research Team, Montigny-Le-Bretonneux, France; 13 Kilimanjaro Christian Medical University College, Moshi, Tanzania; 14 College of Health Sciences, Qatar University, Doha, Qatar; 15 MathematicModelling of Infectious Diseases Unit, Institut Pasteur, Université Paris Cité, UMR 2000 CNRS, Paris, France

**Keywords:** Viral infection, Epidemiology, Health policy

## Abstract

The first vaccine against chikungunya virus (CHIKV) has now been licensed; however, due to a limited knowledge of the underlying global burden, its potential to reduce disease burden remains unknown. We used data from seroprevalence studies, observed cases and mosquito distributions to quantify the underlying CHIKV burden in 180 countries and territories, and we explored the potential impact of vaccination campaigns. We estimate that 104 countries have experienced CHIKV transmission, covering 2.8 billion people, and that, in epidemic settings, the mean duration between outbreaks is 6.2 years, with 8.4% of the susceptible population infected per outbreak. Globally, there are 35 million annual infections, mainly in Southeast Asia, Africa and the Americas. Assuming a vaccine efficacy against disease of 70% and a protection against infection of 40%, vaccinating 50% of individuals over 12 years of age in places and times where the virus circulates would avert 4,436 infections, 0.34 deaths and 37 disability-adjusted life years per 100,000 doses used. These findings highlight the global burden of chikungunya and the potential of CHIKV vaccination campaigns.

## Abstract (web summary)

Estimates of chikungunya virus prevalence across 180 countries and territories show that Southeast Asia, Africa and the Americas carry the most substantiative burden, which would require reactive vaccine campaigns against new outbreaks and routine immunization in heavily endemic areas to reduce transmission.

## Main

Chikungunya virus (CHIKV) belongs to the Alphavirus genus of the Togaviridae family and is primarily transmitted by *Aedes aegypti* and *Aedes albopictus* mosquitoes through human-amplified cycles, with other mosquito species being important in enzootic transmission^1,2^. Infection in humans is characterized by acute symptoms of rash and fever. In addition, approximately 50% of detected acute cases have been estimated to develop chronic joint pain that can last for many months^3,4^. Chikungunya affects individuals of all ages, including those without underlying health complications. Approximately one in 1,000 cases results in death, mainly in neonates, infants and the elderly^5,6^. Cases of chikungunya have been found throughout tropical and subtropical countries around the world^7^. In many places, CHIKV transmission consists of outbreaks followed by periods without circulation^8–10^. However, endemic transmission has also been reported^8,9,11^. After decades with few effective tools to combat CHIKV, substantial investment by the Coalition of Epidemic Preparedness Innovations (CEPI) has led to licensure in the United State and the European Union by the Food and Drug Administration (FDA) and the European Medicines Agency (EMA) of the first CHIKV vaccines, IXCHIQ (VLA1553), developed by Valneva, and VIMKUNYA, developed by Bavarian Nordic^12,13^. Due to the unpredictable nature of CHIKV epidemiology, licensure was obtained through a correlate of protection rather than traditional phase 3 trials.

The vaccine has the potential to reduce the individual burden from CHIKV infection and to protect populations from the economic impact of outbreaks, which can be considerable due to the long-lasting and debilitating nature of chronic sequelae^14^. A major hurdle in the optimal deployment of the vaccine is the limited understanding of the underlying burden from CHIKV around the world and, in addition, how best to deploy the vaccine. The decision whether to use a vaccine typically relies on a vaccine investment case, which quantifies the impact of using a vaccine on the number of infections, cases and deaths averted. However, in the case of CHIKV, there is poor understanding of where the virus circulates, hampering the development of investment cases. The Gavi Alliance, which helps lower-income countries purchase vaccines, has placed CHIKV vaccines on a Learning Agenda, which means that it does not feel there is sufficient information available to make informed decisions on the likely impact of the vaccine^15^. This knowledge gap is driven by frequent clinical misdiagnosis with other pathogens, such as dengue or influenza, and limited access to confirmatory testing^16^. Furthermore, it is unclear whether the epidemic nature of the virus means that the vaccine could be deployed from stockpiles in response to detected outbreaks rather than the integration into immunization schedules.

To quantify the global burden of CHIKV and the potential impact of vaccines, we conducted a literature review to identify countries that have detected outbreaks and used existing knowledge on the global distribution of *Aedes* mosquitoes to identify additional affected countries and the underlying size of the population at risk. We then used mathematical models applied to age-stratified seroprevalence studies to quantify the number of annual infections, cases and deaths per country^17^. This allowed us to critically assess the potential impact of vaccination campaigns to avert future infections, cases and deaths, providing an evidence base to guide future vaccine deployment.

## Results

### Evidence of circulation and proportion of population at risk

**Fig. 1:**
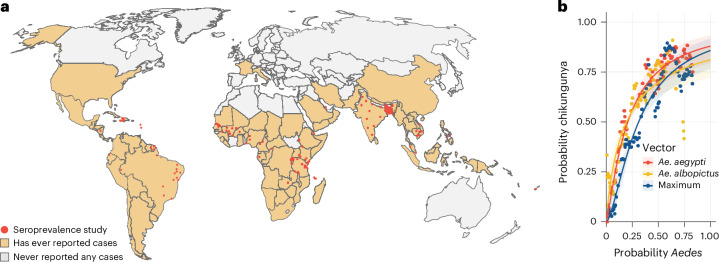
Local circulation of CHIKV and association with vector presence. **a**, Countries with a history of CHIKV transmission and location of seroprevalence studies. **b**, Relationship between average estimated occurrence of *A. aegypti* and *A. albopictus* in a country with the probability that CHIKV has ever been reported in that country. The probability of *Aedes* occurrence was calculated using existing estimates of mosquito distributions in 5-km × 5-km grid cells throughout the world^19^. For each country, we calculated the human population-weighted average occurrence in the country (so mosquito levels in high density areas provided more weight than low density locations). The plot shows the proportion of countries that had ever reported CHIKV outbreaks for locations with a similar level of *Aedes* occurrence. We separately considered *A. aegypti*, *A. albopictus* and a ‘Maximum’, which is the maximum of either *A. aegypti* or *A. albopictus* in any location. The lines present the fit of a logistic regression model.

We conducted a literature review to identify countries and territories (later referred to as countries) that have ever experienced local chikungunya transmission. We identified 140 references that reported CHIKV transmission from 94 countries, representing 52% of all locations considered (Fig. 1a and Supplementary Table 1, 2 and 4). We found that whether or not a country had ever reported CHIKV transmission was strongly correlated with the population-weighted estimated presence of *A. aegypti* (Pearson correlation: 0.89) and *A. albopictus* (Pearson correlation: 0.82) in the country (Fig. 1b)^18^.

**Fig. 2:**
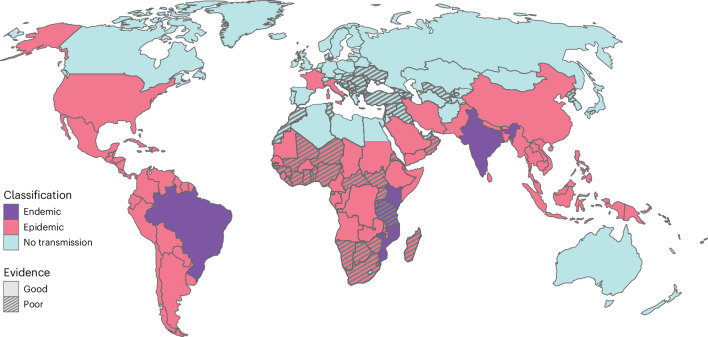
Map of endemic and epidemic countries and level of evidence defined as follows. Endemic: CHIKV circulates persistently on the territory, with infections occurring every year. Epidemic: CHIKV circulates sporadically on the territory, with outbreak being followed by years without detected circulation. No transmission: CHIKV does not circulate on the territory. Good evidence: Epidemiological data from disease surveillance and/or serological data and/or entomological data provide robust evidence for given classification. Poor evidence: Epidemiological, serological and entomological data do not provide robust evidence for given likely classification.

We next divided countries into either ‘endemic’ (with evidence of local transmission occurring each year) or ‘epidemic’ (with outbreaks occurring on a less frequent basis), alongside an assessment of the strength of the evidence. The criteria we used to define epidemic and endemic are set out in Table 1. We categorized six countries as having endemic transmission: four in East Africa (Kenya, Mozambique, Rwanda and Tanzania), one in South America (Brazil) and one in Asia (India). We categorized a further 98 countries as epidemic (Fig. 2 and Extended Data Table 1). Of these, nine (Cape Verde, East Timor, Eritrea, the Gambia, Guinea-Bissau, Côte d’Ivoire, South Sudan, Swaziland and Togo) have never reported cases, but they have high levels of the vectors and have neighboring countries where transmission has been reported. We found no evidence of transmission in 76 countries.

**Table 1:** Criteria used to define epidemic status by country. Confirmed cases are those that are either PCR or IgM confirmed

Epidemic status	Criteria
Endemic - Good evidence	Confirmed autochthonous cases each year for the past 5 years. In the Americas, which has better disease surveillance, we include a criterion for a minimum of 1,000 detected autochthonous cases per year.
Endemic - Poor evidence	Confirmed autochthonous cases each year in at least 3 of the past 5 years and/or serological evidence of endemic transmission. In the Americas, which has better disease surveillance, we include a criterion for a minimum of 100 detected cases per year.
Epidemic - Good evidence	Confirmed cases since 2010 OR Serological evidence of transmission since 2010
Epidemic - Poor evidence	Confirmed cases prior to 2010 OR serological evidence of transmission prior to 2010 OR In low HAQ countries, no confirmed cases but probability *albopictus/aegypti* mosquitoes present >0.5
No transmission - Good evidence	In high HAQ countries, no confirmed cases OR Everywhere else, no confirmed cases and probability *albopictus/aegypti* mosquitoes present <0.1
No transmission - Poor evidence	No confirmed cases and probability *albopictus/aegypti* mosquitoes present 0.1–0.5

**Extended Data Fig. 1:**
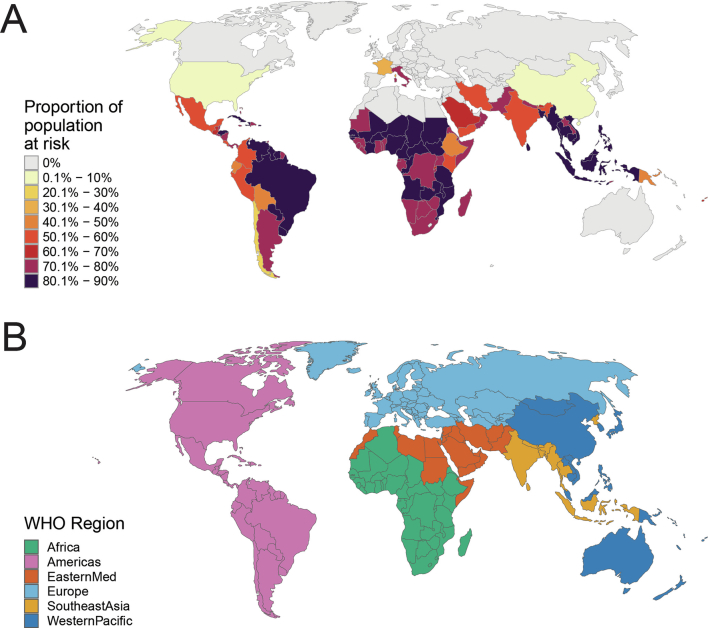
Maps of population at risk and WHO regions. **(a)** Proportion of population at risk per country **(b)** WHO region per country.

Using maps that characterize the distribution of the world’s population^19^, the distribution of *A. aegypti* or *A. albopictus*^18^ and our estimated association between vector presence and CHIKV outbreak risk (Fig. 1b), we estimated that 2.8 billion people globally live in locations at risk of CHIKV transmission (Extended Data Fig. 1).

### Global burden estimates

**Extended Data Fig. 2:**
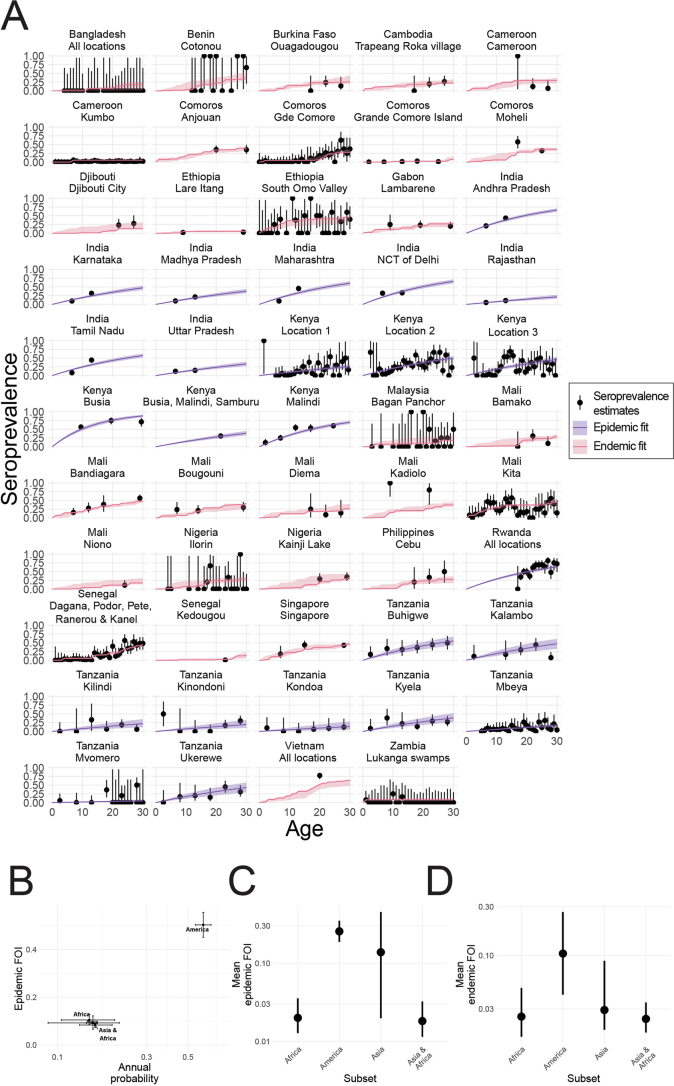
Fits of serocatalytic model and continent-specific estimates for transmission dynamics. **(a)** Seroprevalence data and model fit. For seroprvevalence data, center point is the proportion of seropositive individuals per age group and error bars is the 95% Confidence Interval from a binomial distribution. For the model fit, center line is the model fit using the median of the posterior, ribbons denote the 95% quantile of the posterior distribution. Estimates and 95%CI for different subset of continents from **(b)** the epidemic serocatalytic model **(c)** the endemic serocatalytic model fitted on data from epidemic countries **(d)** the endemic serocatalytic model fitted on data from endemic countries. For panels (**B**), (**C**) and (**D**) center points are the estimated median and error bars the estimated 95% quantile of the posterior distribution.

To estimate the average dynamics of CHIKV transmission in endemic and epidemic countries, we next fit serocatalytic models to the results of 49 age-specific seroprevalence studies collected from 29 countries (Extended Data Fig. 2 and Supplementary Table 2). We found that in endemic locations, the mean annual probability of infection among the susceptible population was 0.024 (95% confidence interval (CI): 0.018–0.035), ranging from 0.0017 to 0.074 across the 24 different endemic locations in our datasets. In epidemic settings, the mean annual probability of infection was 0.016 (95% CI: 0.013–0.024), ranging from 0.0004 to 0.065 across locations. However, we found that, on average, there was no transmission in most years, with an annual probability of an outbreak occurring of 0.16 (95% CI 0.13–0.20), equivalent to a duration of 6.2 years between outbreaks (95% CI: 5.1–7.6). We estimated that the mean percentage of the susceptible population that gets infected during outbreaks was 8.4% (95% CI: 7.2–9.1%).

**Fig. 3:**
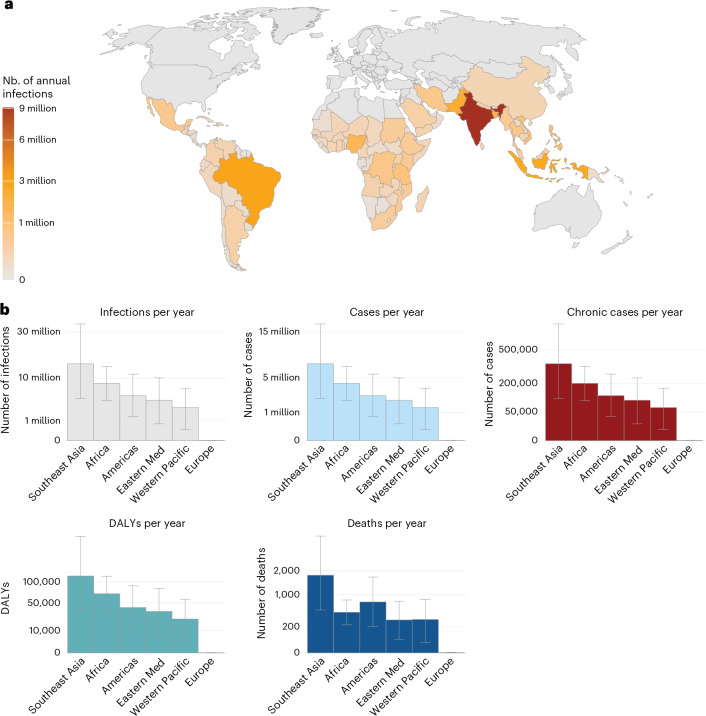
Burden estimates. **a**, Annual infections per country. **b**, Annual infections, cases, chronic cases, DALYs and deaths per WHO region. Error bars represent 95% CIs. Eastern Med, Eastern Mediterranean; Nb., number.

Using the estimated force of infection (FOI), we calculated the average number of infections occurring annually within each country. We estimated that, globally, there are 35,300,000 infections per year (95% CI: 20,900,000–56,500,000). The most affected World Health Organization (WHO) region is Southeast Asia, followed by Africa and the Americas (Fig. 3a, Extended Data Fig. 1 and Supplementary Table 3). We estimated that there are 13,800,000 infections in endemic countries, driven largely by India (9,100,000 annual infections) and 21,600,000 infections in epidemic countries (Supplementary Table 4). We estimated that these infections lead to 17,700,000 symptomatic cases, 848,000 with chronic sequelae and 3,700 deaths (95% CI: 2,100–6,100)^17^. Overall, we estimated that there are 284,000 disability-adjusted life years (DALYs) lost to CHIKV each year (Fig. 3b).

### Potential vaccine impact

**Extended Data Fig. 3:**
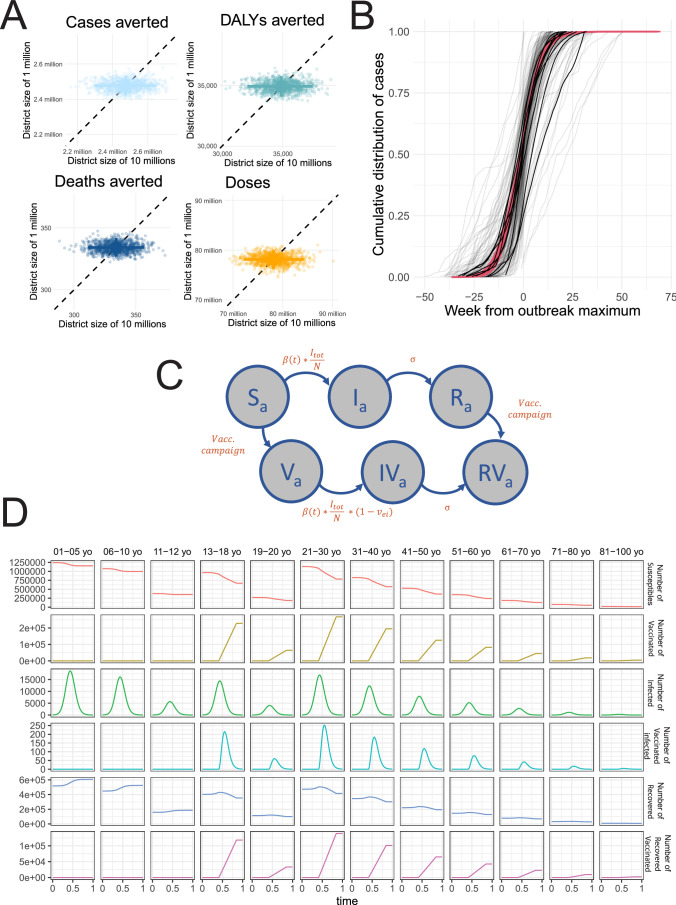
Details on the simulation framework. **(a)** Sensitivity analysis on the impact of the population size on the districts where the simulated outbreak occurs. Center bar denote the average value out of 1000 simulations and error bars contain 95% of simulated outputs. **(b)** Shape and timing of observed outbreaks against simulated outbreaks. Empirical distribution function of chikungunya cases reported in Brazil since 2015 by state (thin black lines) and nationally (plain black lines). Empirical distribution function of simulated outbreaks with varying force of infection (red lines). **(c)** Structure of the SIRV model for a given age group **(d)** Outbreak simulated for the base case vaccination scenario over one year in a representative population of 10 million people. (Abbreviations: **S**: Susceptibles; **V**: Vaccinated; **I**: Infected; **IV**: Infected Vaccinated; **R**: Recovered; **RV**: Recovered Vaccinated).

Using our estimates of the underlying level of infection, we estimated the potential population impact of a vaccine using a transmission model, using IXCHIQ as a case study. As there are currently no existing measured estimates of the efficacy of IXCHIQ, we relied on an expert panel consisting of individuals from academia, WHO, CEPI and Gavi to obtain consensus estimates of key characteristics of the vaccine (Extended Data Table 2). The panel decided on the assumptions that the vaccine provides 70% protection against disease and 40% protection against infection and an average period of protection of 5 years. For endemic settings, we assumed that vaccines were introduced to individuals 12 years of age and older based on current age-specific recommendations through an initial campaign followed by supplemental immunization campaigns occurring every 5 years. We assumed that a set proportion of the population is vaccinated each campaign, irrespective of prior vaccination status. For epidemic settings, we considered the use of a vaccine stockpile where vaccines are distributed in response to an outbreak in a district of 10 million inhabitants, with a delay for the outbreak to be detected (based on a minimum number of cases occurring), and that vaccination occurs over a set duration of time. We assumed that transmission is seasonal. To ensure that the timings between the reactive campaigns and the start of the outbreak were realistic, we calibrated the simulated outbreak to epidemic time series retrieved from the Brazilian national case notification database (Extended Data Fig. 3). The trajectory of each outbreak and, in particular, when the epidemic ends depended on contributions of immunity from the vaccines, immunity from infections and seasonality in the FOI.

**Fig. 4:**
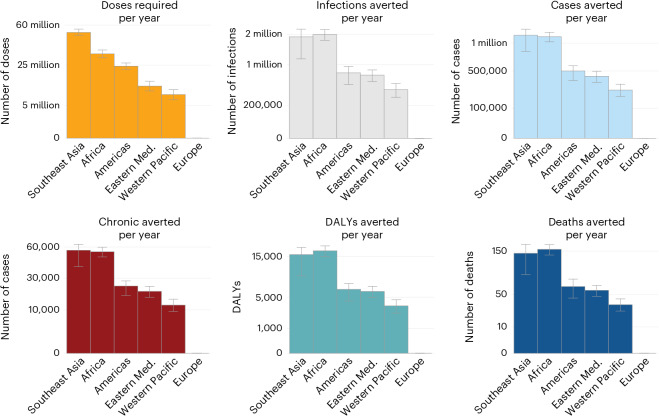
Summary of impact by WHO region for the base case model. Error bars represent 95% CIs. Eastern Med., Eastern Mediterranean.

We found that, on average, achieving 50% vaccination coverage of the population exposed to an outbreak would require 132 million doses per year (53.9 million in endemic locations and 78.0 million in epidemic locations). The total number of doses was strongly driven by India, where CHIKV circulates endemically (33 million doses per year). We estimated that this level of vaccination would lead to 5.8 million fewer infections, 168,000 fewer chronic cases, 450 fewer deaths and 48,500 DALYs averted per year (Fig. 4 and Supplementary Table 3). On average, globally, per 100,000 doses used, we estimate 4,400 (95% CI: 3,800–4,800) infections, 2,700 (95% CI: 2,400–2,800) cases, 0.35 (95% CI: 0.30–0.37) deaths and 37 (95% CI: 32–40) DALYs averted. The impact in epidemic settings was higher than in endemic settings with 5,500 (95% CI: 5,000–6,000) infections averted per 100,000 doses used in epidemic settings compared to 3,000 (95% CI: 1,600–3,400) in endemic settings.

**Fig. 5:**
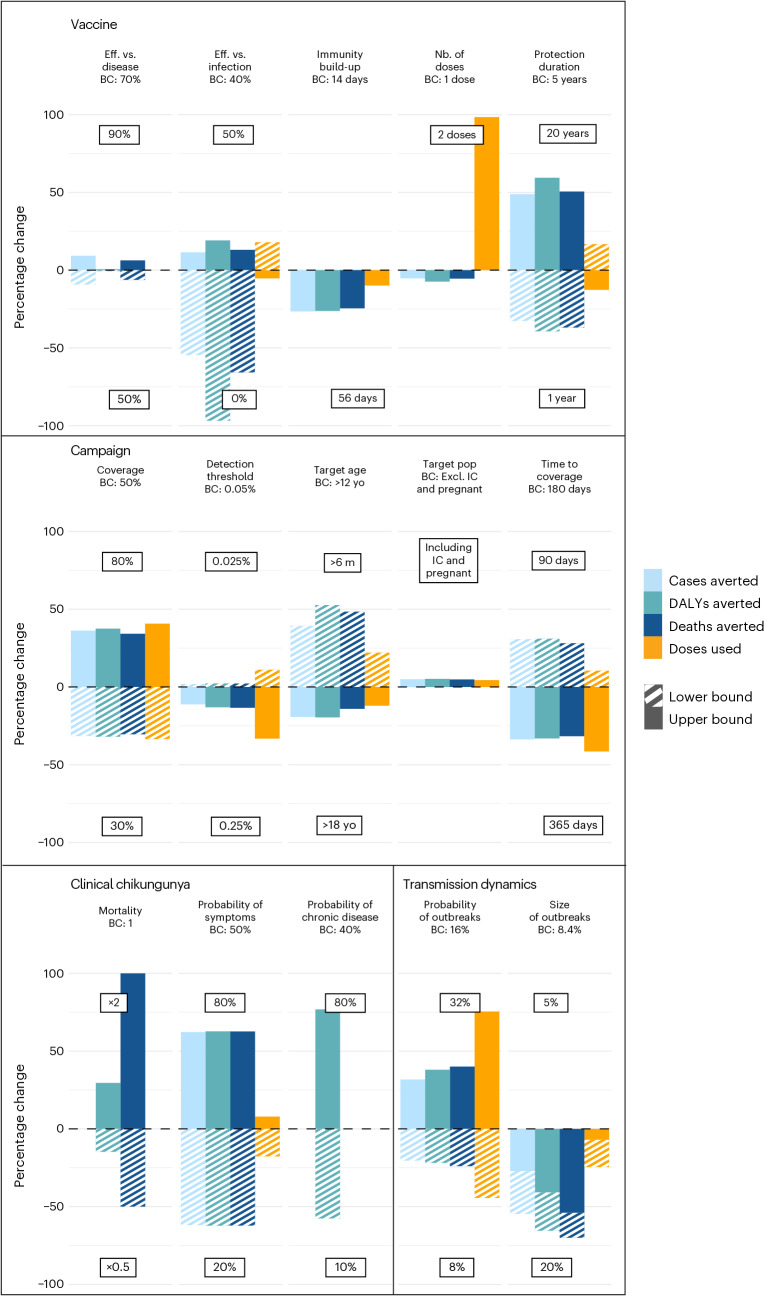
Sensitivity analysis of the model parameters. Percentage change in doses used, cases, DALYs and deaths averted when changing one parameter compared to the base case (BC) scenario. Eff., Efficacy; Excl., excluding; IC, immunocompromised; m, months; Nb., number; pop., population; Prob., probability; yo, years old.

The model results are reliant on several strong assumptions linked to vaccine, epidemiological and rollout characteristics. To explore the relative importance of each assumption, we varied each parameter in turn and compared our estimates of the number of infections, cases, deaths and DALYs with those estimated in the base case for epidemic scenarios (Fig. 5). We found that the conclusions from the model are most sensitive to a range of assumptions about the vaccine characteristics (effectiveness against infection and disease and duration of protection), the rollout strategy (coverage level, time to coverage and time taken to detect outbreaks) and the natural history of disease (probability of symptoms). Decreasing the size of subdistricts where a simulated outbreak occurs did not change the average estimates for burden at the country level (Extended Data Fig. 3). We note that both increasing and decreasing the size of outbreaks can lead to reductions in the impact of the vaccine. This is due to the complex interactions between immunity from vaccines and immunity from natural infection. We found that continent-specific estimates suggest that the dynamics are broadly similar across affected regions (Extended Data Fig. 2). The only exception is South America, which had a higher FOI than elsewhere. However, CHIKV is new to this continent, and, as the virus now encounters substantial population immunity, we can expect the FOI to fall.

## Discussion

Here we have presented an overview of the global burden from CHIKV infection and the potential benefits from vaccination campaigns. Our findings demonstrate that large portions of the world are at risk from the pathogen, usually from sporadic outbreaks separated by approximately 6 years. Our findings suggest that reactive vaccine campaigns using a pre-existing stockpile could substantially reduce the burden from CHIKV. In heavily endemic areas, such as India and Brazil, routine immunization would also reduce the impact of the virus.

Tackling CHIKV infections using the vaccine will require integration of the vaccine into existing immunization protocols. However, for many parts of the world, this is unlikely to be an attractive prospect if epidemics are infrequent and the duration of vaccine protection remains poorly understood. In this context, responsive vaccine campaigns from stockpiles are a good alternative, as is done with cholera^20^. However, a stockpile-based approach relies on being able to successfully detect outbreaks and then appropriately respond in a timely manner. We showed that, with a delayed response, where a deployment occurs only after thousands of infections have occurred, the vaccine can still limit the size of the outbreak and the burden from the pathogen. However, with further delays, the potential impact of a vaccine campaign is reduced. Furthermore, in many settings, such as in the Philippines or in Burkina Faso, it has been shown that multiple large CHIKV outbreaks have occurred without a single case reported^8,9^. Improved case detection will be required in many countries, especially in Africa and Asia, to go alongside any stockpile-based approach. Finally, even with the timely detection of outbreaks, there will be important practical challenges in implementing large-scale vaccine stockpiling campaigns that cannot be ignored.

Alongside the ability to detect outbreaks, the optimal vaccine strategy will depend on the specific epidemiology of a location. We relied on a simplified approach that divided countries into ‘epidemic’ and ‘endemic’, with epidemic countries implementing reactive vaccine campaigns and endemic countries using continuous immunization programs. However, there will be substantial heterogeneity within a country. It is clear, for example, that, within Brazil and India, there are areas that experience sporadic epidemic transmission, whereas other areas will have more sustained transmission. It may be that mixed strategies are possible, with routine immunization in some areas and stockpile-based approaches in others, as is done with yellow fever vaccines in Brazil. The feasibility of such an approach will depend on vaccine deployment infrastructure and other political and budgetary considerations.

We estimate that, in locations where CHIKV circulates, an average of 1.5% of the susceptible population gets infected each year. These estimates are largely consistent with findings from a previous systematic review that estimated a median FOI of approximately 0.7% (ref. ^17^). Our findings of 17.8 million annual symptomatic cases is around a fifth of the estimated global burden of symptomatic dengue virus (DENV), despite the same areas of the world affected^21^. The differences in CHIKV and DENV burden appear linked to differences in underlying epidemiological patterns, with DENV more capable of transitioning to sustained endemic circulation than CHIKV. The reasons for these inherent differences remain unclear, although they may include underlying differences in the tendency to cause symptoms, different viral dynamics within the mosquito and the possibility of reinfection by different DENV serotypes and associated complex patterns of population immunity^22,23^. Climate change is leading to larger areas being suitable for *Aedes* vectors, coupled with longer transmission seasons, and increased mobility in and out of locations with CHIKV transmission will lead to more frequent CHIKV introductions and sustained outbreaks^24^.

Alongside the long-term chronic sequelae, there is a growing realization of the deadly nature of the pathogen^25^. We estimated that over 3,500 lives are lost each year from CHIKV, more than reported. This difference can be accounted for from the substantial under-detection of outbreaks, especially in places where DENV co-circulates, and misattribution of cause of death, especially as most CHIKV deaths occur in the elderly who often have other comorbidities^26^. Our estimates of the disease burden from CHIKV are reliant on assumptions of the probability of symptoms following infection. CHIKV infection is generally considered to result in disease; however, a cohort study in the Philippines with regular blood draws and active disease surveillance found that over 80% of infections were subclinical^27–29^. Heterogeneity in the probability of an infected individual becoming a detected case will be driven by differences in healthcare seeking and surveillance systems. There is also the potential that the probability of symptoms differs by CHIKV lineage^30^.

CHIKV vaccines used a correlate of protection as a means to obtain licensure^31^ rather than from standard phase 3 randomized controlled trials. This means that there do not exist efficacy estimates that would normally be available. In the absence of trial data and long-term antibody data, our expert panel settled on a set of assumptions for the vaccine to inform our model; however, these were ultimately subjective in nature, and there is good reason to think that these assumptions are conservative. IXCHIQ is a live attenuated vaccine, which generates high titers, which remain stable for at least 2 years and probably longer and are associated with protection^32,33^. Natural infection results in persistent titers, lasting decades, and the presence of any antibody titers is associated with protection against infection and disease^27,31,34^. Assuming an improved profile of 90% protection against infection and disease would lead to a near doubling of the impact of the vaccine to 5,270 cases averted per 100,000 doses used (compared to 2,650 cases averted in our base model). We assumed that IXCHIQ will protect against long-term sequelae; however, in the absence of trials, this has not been demonstrated. Phase 4 trials, which are currently planned for Brazil, will help us understand how realistic these key assumptions are. Finally, some side effects have been associated with IXCHIQ^35,36^. In a phase 3 trial, severe adverse events were reported in 2% and arthralgia in 18% of vaccine recipients, although most of the arthralgia was mild, with 0.3% of recipients developing severe arthralgia^36^. We have not included these side effects in the DALY calculations.

Our study is subject to limitations. Our estimates of the underlying epidemiology of CHIKV and the potential impact of the vaccine are necessarily reliant on strong assumptions. In particular, there is limited understanding about chikungunya transmission, disease history and the efficacy of the new vaccines. However, we systematically explored the impact of each key assumption on our estimates, allowing us to identify the specific areas that should be targeted by future studies. We divided all affected countries into either ‘epidemic’ or ‘endemic’ and assumed that the underlying level of transmission is the same across all epidemic countries and that transmission levels are the same across endemic countries. Although this approach will miss the nuances of local transmission dynamics, this simplified methodology provides a tractable and useful description of the overall global risk distribution of a poorly understood pathogen. Country-specific efforts that incorporate local understanding of risk, logistical and budgetary constraints will help tailor national intervention strategies. We attempted to account for within-country heterogeneity in the population size at risk of infection by using the relationship between average national estimates of *Aedes* occurrence and history of CHIKV transmission. The relationship between local risk and national average risk is likely to be more complex but is unlikely to result in substantial differences in overall estimates of national burden. Our estimates of infection risk are based on existing cross-sectional seroprevalence studies. Although we restricted the analysis to data generated using population-representative samples, they will ultimately have been conducted with different protocols and with different assays. There may also be other cross-reactive alphaviruses circulating in the same communities, which would lead to some false positives. We assumed that approximately one in five individuals develop chronic symptoms; however, this remains a poorly understood estimate that is likely to depend on age, sex and CHIKV strain^37^.

In conclusion, CHIKV is a major threat to public health across much of the world. However, with new vaccines, there is a real opportunity to combat this threat. Improved abilities to identify and quickly respond to outbreaks will be central to maximizing the potential of the vaccine.

## Methods

### Countries and territories considered

We considered countries and territories (as defined by the United Nations) with a population size of over 200,000 inhabitants. We included territories to avoid problems with some (especially island) territories being in different CHIKV risk zones than the remainder of the country (for example, French Guiana and mainland France). This resulted in a total of 180 countries and territories.

### Literature review

Taking each country and territory in turn, we used the following resources:

- Google (search term ‘chikungunya AND [COUNTRY]’)
- Google scholar (search term ‘chikungunya AND [COUNTRY]’)
- PubMed (search term ‘chikungunya AND [COUNTRY]’)
- GIDEON
- WHO/PAHO websites
- ProMED (search term ‘chikungunya AND [COUNTRY]’)

We identified Ministry of Health websites where possible. For each country and territory, we identified whether there was evidence of CHIKV autochthonous transmission. We considered evidence of transmission as either the detection of cases (with at least one case confirmed via polymerase chain reaction (PCR) or IgM) or from seroprevalence studies (IgG or IgM). We considered outbreaks from any year prior to 2023. We recorded the specific year of the outbreak when they occurred between the years 2011 and 2022 (Supplementary Table 1).

### Seroprevalence studies

As part of the literature review process, we specifically highlighted seroprevalence studies for CHIKV for data extraction. Our inclusion criteria were studies in healthy individuals from the general population that had tested for CHIKV IgG. We excluded seroprevalence studies in suspected cases (Supplementary Table 2). From each detected study, we identified the location of the study (from which we subsequently identified coordinates), the number of individuals per age group and the number of individuals who tested positive for CHIKV. We subsequently contacted the authors of all identified seroprevalence studies to obtain finer-scale data on age and location. From this process, we obtained 49 age-stratified seroprevalence datasets from research groups covering 97 locations across 29 countries.

### Relationship between *Aedes* mosquito distribution and CHIKV

A previous estimate modeled the presence of *A. aegypti* and *A. albopictus* in 5-km × 5-km grid cells around the world using climate data and a large global repository of mosquito trap data^19^. We explored the extent to which these estimates of *A. aegypti* and *A. albopictus* were associated with CHIKV presence in a country.

We first extracted the human population-weighted average presence (MosqWeighted) of *A. albopictus* and *A. aegypti* in a country *j* using the estimated distribution of population density in the same grid cells as the mosquito data from WorldPop^19,38^.

1 $${\rm{MosqWeighted}}_{j}=\sum _{i}{\rm{pop}}_{i,\;j}{\rm{mosq}}_{i,\;j}/{\rm{POP}}_{j}$$

where ${\rm{pop}}_{i,\,j}$ is the human population in cell *i* in country *j*; ${\rm{mosq}}_{i,\,j}$ is the mosquito presence probability from Kraemer et al.^18^ for that same cell *i*; and ${\rm{{PO}{P}}}_{j}$ is the total population size in country *j*. We used this approach as mosquito levels in places with large population sizes are more relevant to CHIKV burden than places with few inhabitants.

We then compared these estimates of mosquito presence to the probability of that country having ever reported CHIKV transmission by calculating the proportion of countries that had ever reported outbreaks among those with population-weighted mosquito levels of increasing sizes (Fig. 1b). To quantify the relationship between population-weighted mosquito levels and presence of CHIKV, we used logistic regression:

2 $${\rm{{logit}({presenc}{e}}}_{j})={\beta }_{0}+{\beta }_{1}\cdot {\rm{{MosqWeighte}{d}}}_{j}$$

We conducted separate analyses for *A. aegypti* and *A. albopictus* and then a separate one where we just used the greater one of the two species within each cell.

### Estimating the population at risk

For each 5-km × 5-km grid cell of a given country, we used the fitted relationship between mosquito occurrence and the probability of CHIKV outbreaks from logistic regression (Fig. 1b) and the number of people living in that cell to obtain a weighted average of the size of the population at risk in that location.

3 $${\rm{{PopAtRis}{k}}}_{j}=\sum _{i}{\rm{{po}{p}}}_{i,\;j}\cdot {\rm{{ProbOutbrea}{k}}}_{i,\;j}$$

where ${\rm{ProbOutbreak}}_{i,\;j}$ is the estimated probability of observing an outbreak in cell *i* in country *j* based on the fitted logistic model in equation (2), where we use the greater of *A. albopictus* and *A. aegypti* presence within that cell. For India, China and the United States, which have large populations, we added an additional mask that assumes CHIKV transmission occurs only in areas where there is good evidence of sustained transmission, from either national seroprevalence studies (India) or good case reporting (China and the United States)^11^. In masked areas, ${\rm{ProbOutbreak}}_{i,\,j}$ is set to 0.

### Assigning of epidemic status by country

To estimate the reliability of case data for each country, we used the Healthcare Access and Quality (HAQ) Index that is measured on a scale from 0 (worst) to 100 (best) based on amenable mortality^39^. Countries in the first two deciles of the index (HAQ Index > 82.2) were classified as having a good surveillance system^39^. We next used the data on case occurrence, seroprevalence studies and mosquito distribution to categorize the epidemic status of all countries and territories as set out in Table 1. Each country and territory was assigned to ‘endemic’ (that is, with evidence of sustained transmission each year), ‘epidemic’ (that is, evidence of transmission but not sustained across years) or ‘no transmission’. We also provide an assessment of the strength of the evidence for the categorization of each country (Table 1).

### Estimating CHIKV transmission dynamics

We used the collected data to inform different models and get estimates on parameters that capture the global dynamics of CHIKV. These models rely on the definition of FOI, which represents the rate at which the susceptible members of the population in a community get infected. For endemic countries, we estimated an FOI using a serocatalytic model in a Markov chain Monte Carlo (MCMC) framework. In epidemic countries, we considered that FOI was not constant and estimated an annual probability of an outbreak occurring as well as the annual FOI when an outbreak occurs.

We subsetted the serological datasets based on the status classification of the country that they originate from. The epidemic-prone countries were used to fit a single epidemic model to estimate an overall probability of outbreak occurrence (*μ*) and average outbreak size (λ). The endemic countries were used to fit a single endemic model to estimate the distribution of time-constant FOIs across locations characterized by its mean (*λ*_endemic_) and standard deviation (*σ*_endemic_).

### Epidemic model

We assume that outbreaks have a yearly probability *μ* of occurring with an average size of *λ* and a standard deviation σ that are the same across locations. In each location, we simulated outbreak patterns and retained the global parameters that generated the most likely patterns. An outbreak pattern in a given location is suitable if it approximates well age-specific cumulative FOIs inferred from the age-stratified seroprevalence in that location.

For a given location *r* and a given age group *A*, we call ${N}_{{pos},{rA}}$ the number of samples that tested positive for IgG antibodies against CHIKV and ${N}_{{tot},{rA}}$ the total number of samples tested. Note that the size of the age groups differed from one location to another. We use a binomial likelihood to fit our model to the data:

$${N}_{{pos},{rA}} \sim {\rm{Binomial}}({N}_{{\rm{{tot}}},{rA}},{p}_{{rA}})$$

with ${p}_{{rA}}$ being the probability that an individual from a given age group *A* will have been infected by the time the sample was collected. By definition, the probability *p* of being infected at age *a* follows an exponential law of rate equal to the FOI (escaping infection from birth to age *a*). And the *p*_*rA*_ (proportion of people who have been infected from birth to age *a*) is the cumulative distribution function of *p*. Taking into account grouping by age, we get:

$${p}_{rA}=1-{e}^{-{\underline{\varGamma }}_{rA}}$$

with ${\underline{\varGamma }}_{rA}$ being the average cumulative FOI for an age group *A* that we estimated to be the mean value of the cumulative FOI Γ_*rN*_ for all ages included in the year group:

$${\underline{\varGamma}}_{rA}={\rm{mean}}_{N\in [{\rm{age}}_{\rm{min}};{\rm{age}}_{\rm{max}}]}({\varGamma}_{rN})$$

From their birth to the study year, any individual who is *N* years old will have been exposed to the cumulative FOI:

$${\varGamma }_{{rN}}=\mathop{\sum }\limits_{i=0}^{N}{\lambda }_{{ri}}$$

with *λ*_*ri*_ being the annual FOI in location *r* at year (*Y* − *i*) where *Y* is the year where the serum samples were collected in this location.

*λ*_*ri*_ was simulated during an iterated filtering process drawn from *Beta*(*α*, *β*) with a probability *μ* and set to 0 otherwise. *α* and *β* were calculated such that the mean and standard deviation of the distribution were equal to *λ* and σ. For parameter identifiability purposes, σ was fixed at 0.0025. The parameters estimated are *μ* and *λ* using a sequential Monte Carlo (SMC) Bayesian framework embedded in C and accessed using R and the ‘pomp’^40^ package. Uninformative uniform priors *U*(0,1) were used for *λ* and σ. In total, 1,000 particles divided into equal-sized blocks (one by location) were used to simulate outbreak patterns and explore the parameter space. Each particle filtering instance was replicated 20 times to estimate the likelihood and associated uncertainty.

### Endemic model

Following the previous notations, we assumed that outbreaks occur every year and estimated the average FOI resulting from that endemic pattern. We assumed all the *λ*_*ri*_ to be equal to *λ*_*r*_ such that

$${\varGamma }_{{rN}}=N* {\lambda }_{r}$$

where *λ*_*r*_ is the time-constant FOI estimated for location *r*. *λ*_*r*_ is drawn from a beta distribution *Beta*(*α*, *β*) of mean *λ*_endemic_ and standard deviation *σ*_endemic_. The hyperparameters *λ*_endemic_ and *σ*_endemic_ were also estimated in our model using uninformative uniform priors *U*(0,1) for both. We used an MCMC Bayesian framework using Stan and R. The posterior distribution of considered parameters was sampled using four chains of 4,500 iterations each including a burn-in phase of 500 iterations.

### Vaccine simulation framework

For each country, we simulated transmission of chikungunya. If a country has more than 10 million inhabitants, we considered that it was structured with several 10-million population subdistricts with independent FOI patterns from one to another.

The population has an age structure derived from the 2020 United Nations World Population Prospects^41^. They were grouped in the 12 following age groups: 0–5, 6–10, 11–12, 13–18, 19–20, 21–30, 31–40, 41–50, 51–60, 61–70, 71–80 and 81+. We used these age groups to allow for age-specific vaccine policies (especially where we consider 12+ and 18+ vaccination strategies) as well as to allow sufficient granularity to have age-specific mortality.

Each age group has six different compartments that individuals can be allocated to depending on their infection and vaccination status:

- S: unvaccinated, never-infected individuals
- I: unvaccinated, infectious individuals
- R: unvaccinated, recovered individuals (seropositives)
- V: vaccinated never-infected individuals
- IV: vaccinated, infectious individuals
- RV: vaccinated, recovered individuals (seropositives)

We ran the simulation for a period of 20 years, during which we measured the impact estimates.

Prior to the first year, the FOIs for each year were drawn. If the country is endemic, a time-constant FOI was drawn from a Beta distribution of mean *λ*_endemic_ and standard deviation *σ*_endemic_. If the country is epidemic, for each year, the annual FOI was set to 0 (no transmission event) with probability (1 − *μ*) and to a location-specific FOI drawn from a Beta distribution of mean *λ*_endemic_ and standard deviation *σ*_endemic_ with probability *μ*.

Every year, the following events occur:

- Loss of vaccination protection
- Running a deterministic SIRV model with seasonal transmissibility calculated from the FOI previously drawn
- Aging of the population

### Loss of vaccination

The proportion of vaccinated people in the population exponentially decays with a decay rate ${v}_{\rm{dur}}^{-1}$ where *v*_dur_ (base case 5 years) is the vaccine duration of protection. This is translated by a flow of individuals from compartment V to S and from compartment RV to R.

### Vaccination schedule

In endemic countries, the vaccination campaign occurs every 5 years and vaccinates 50% of the unvaccinated population (S + R) over 180 d. Vaccinations occur at a fixed daily rate over the duration of the vaccination campaign.

In epidemic countries, vaccination starts as soon as a threshold number of cases is detected (500 cases per million) and also aims at vaccinating 50% of the unvaccinated population (S + R) over 180 d. However, vaccination can stop prematurely if the outbreak dies out reaching 50 daily cases per million.

We assume the vaccine to be delivered in a single dose, with a time to reach protective immunity of 14 d. The vaccine provides a protection against infection of 40% and protection against disease of 70%.

### SIRV model

The chikungunya transmission and vaccination of the population are simulated simultaneously using an age-stratified SIRV model described by the following system of differential equations for a given age group *a* (Extended Data Fig. 3):

$$\frac{{\rm{d{S}}}_{a}}{{\rm{d}}{t}}=-{St}{I}_{a}-{St}{V}_{a}$$

$$\frac{{\rm{d}}{V}_{a}}{{\rm{d}}{t}}={St}{V}_{a}-{VtI}{V}_{a}$$

$$\frac{{\rm{d}}{I}_{a}}{{\rm{d}}{t}}={St}{I}_{a}-{It}{R}_{a}$$

$$\frac{{\rm{d}}{{IV}}_{a}}{{\rm{{d}}}{t}}={Vt}{{IV}}_{a}-{IVt}{{RV}}_{a}$$

$$\frac{{\rm{d}}{R}_{a}}{{\rm{d}}{t}}={It}{R}_{a}-{RtR}{V}_{a}$$

$$\frac{{\rm{d}}{{RV}}_{a}}{{\rm{{d}}}{t}}={IVt}{{RV}}_{a}+{RtR}{V}_{a}$$

with:

- $$S{{tI}}_{a}=\beta (t)* \frac{{I}_{\rm{tot}}}{N}* {S}_{a}$$
- $${VtI}{V}_{a}=\beta (t)* (1-{v}_{{ei}})* \frac{{I}_{\rm{tot}}}{N}* {V}_{a}$$
- $${It}{R}_{a}=\sigma * {I}_{a}$$
- $${IVt}{{RV}}_{a}=\sigma * {{IV}}_{a}$$
- ${St}{V}_{a}$ and ${RtR}{V}_{a}$ being dictated by the daily rates of vaccination as described previously
- ${I}_{\rm{tot}}=\sum _{a}({I}_{a}+I{V}_{a})$, the total number of infected individuals
- $N=\sum _{a}({S}_{a}+{V}_{a}+{I}_{a}+I{V}_{a}+{R}_{a}+R{V}_{a})$, the total population size
- *v*_*ei*_ is the vaccine-induced protection against infection
- 1/*σ* is the mean duration of infectiousness
- *β*(*t*) is the seasonal transmission rate, linearly decreasing over time (sawtooth pattern with an offsetted baseline)

### Population aging

Each year, individuals from compartments of age *a* go to compartments of age *a* + *1*. A new birth cohort of completely susceptible individuals is introduced in the population at age 0, and individuals in the last age compartment (100+) are removed from the population. To keep the age structure constant through time, each compartment is then proportionally adjusted.

### Model parameters

As there is considerable uncertainty in the vaccine characteristics and feasibility of different deployment strategies, there was a meeting of experts convened, with representatives from WHO, CEPI, Gavi and academia, where a broad consensus was reached. These are summarized in Extended Data Table 2.

**Extended Data Fig. 4:**
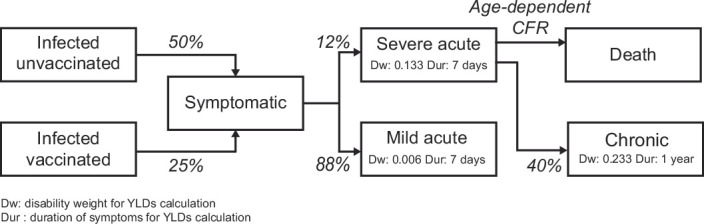
Burden diagram. Summary diagram for YLDs calculation. (Abbreviations: CFR: Case Fatality Ratio; Dw: Disability weight; Dur: Duration; YLDs: Years lived with disability).

Unvaccinated infections have a 50% chance of being symptomatic (expert opinion), and vaccinated infections have 50% × (1-vaccine protection against disease) / (1-vaccine protection against infection) = 50% × (1−0.7) / (1−0.4) = 25% chance of developing symptoms. We assume that all symptomatic infections are detected by the surveillance systems (number of cases). (Extended Data Fig. 4).

Symptomatic infections have a 88% chance of having a mild acute phase and a 12% chance of having a severe one^6,42,43^. Severe acute phases have then a 40% chance of developing chronic symptoms (arthralgia)^44–47^. We assume the acute phases to last for 7 d on average and chronic symptoms to last for 1 year^3,37,48^.

The probability of death given symptomatic infection (case fatality rate (CFR)) is age dependant and was based on clinical data from Paraguay^6^.

Years lived with a disability (YLDs) were computed by assuming a disability weight of 0.006 for mild acute symptoms, 0.133 for severe acute symptoms and 0.233 for arthralgia (chronic symptoms)^49–54^. Years of life lost (YLLs) were measured as being the difference between the age of death and the mean life expectancy of the country. Individuals dying after the mean life expectancy of the country did not contribute to the YLL calculation (Extended Data Fig. 4).

By definition, DALYs lost to chikungunya were calculated as being the sum of YLLs and YLDs.

### Statistical analysis

Correlation coefficients between the probability of local transmission and the probability of vector presence were computed using the Pearson correlation coefficient. Seroprevalence estimates in a specific group were computed as the proportion of seropositive individual in that group, and CIs were computed as a binomial proportion CI using the Wilson score interval. Information on the Bayesian frameworks used, including choice of priors and algorithm parameters, are detailed in specific sections of the Methods. The estimates and 95% CI of the serocatalytic models are the median of the sampled posteriors along with their 2.5% and 97.5% quantiles used for the 95% CIs. Results and 95% CI on the burden estimates and simulated vaccine impact are the mean and the 2.5% and the 97.5% quantiles calculated from the outcome of 1,000 bootstrapped simulations.

### Inclusion and ethics statement

This project is a result of a collaboration among several institutions, including from many countries that are impacted by CHIKV and are, hence, directly concerned by the presented results. This study will be relevant by providing estimates of how much of this burden can be avoided locally by implementing vaccination campaigns. All co-authors contributed right from the early stages of the project. All models were developed using anonymized datasets. The data were either extracted from existing publications or provided by the underlying data collectors without any personal identifiers. The datasets are credited to the local research groups that generated them. Local and regional research was duly acknowledged in citations. As there are currently no existing measured estimates of the efficacy of the IXCHIQ vaccine, consensus estimates of key characteristics of the vaccine were obtained from an expert panel consisting of individuals from academia, WHO, CEPI and Gavi.

### Reporting summary

Further information on research design is available in the Nature Portfolio Reporting Summary linked to this article.

## Online content

Any methods, additional references, Nature Portfolio reporting summaries, source data, extended data, supplementary information, acknowledgements, peer review information; details of author contributions and competing interests; and statements of data and code availability are available at 10.1038/s41591-025-03703-w.

## Supplementary information

Reporting Summary

Supplementary Table 1

Supplementary Table 2

Supplementary Table 3

Supplementary Table 4

## Figures and Tables

**Extended Data Table 1:**
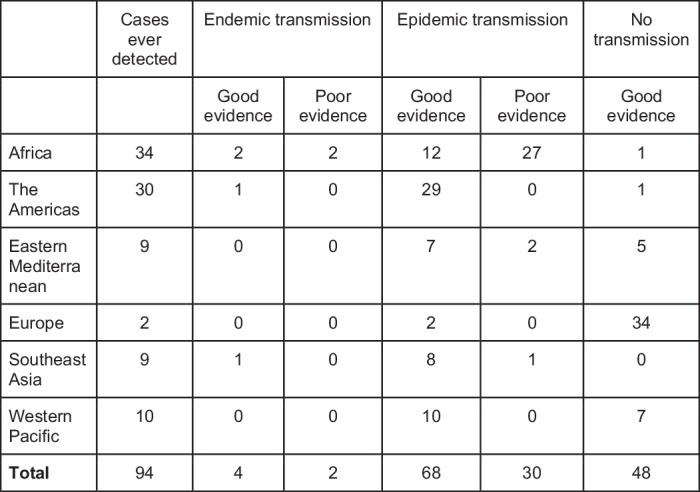
Country classification by WHO region

Number of countries and territories by epidemic status by WHO region.

**Extended Data Table 2:**
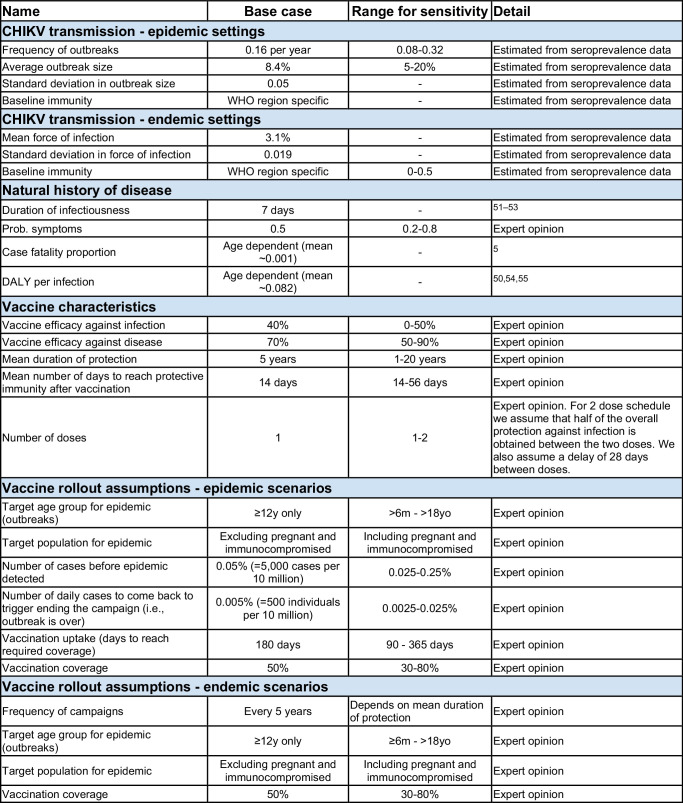
Parameters used

Model parameters for CHIKV epidemiology, natural history of disease, vaccine characteristics and rollout assumptions and ranges used for sensitivity analysis.

## Data Availability

The publicly available seroprevalence datasets will be made available on GitHub, along with the references of their article of origin where relevant. Links and references to publicly available data can be found in Supplementary Table 1 for case reports and in Supplementary Table 2 for seroprevalence studies.

## Code availability

All the code used for the analysis presented here is publicly available in the following GitHub repository: https://github.com/G-ribeiro-dos-santos/chik-global-burden.
