# Author Correction: Global burden of chikungunya virus infections and the potential benefit of vaccination campaigns

**DOI:** 10.1038/s41591-025-04065-z

**Published:** 2025-11-10

**Authors:** Gabriel Ribeiro dos Santos, Fariha Jawed, Christinah Mukandavire, Arminder Deol, Danny Scarponi, Leonard E. G. Mboera, Eric Seruyange, Mathieu J. P. Poirier, Samuel Bosomprah, Augustine O. Udeze, Koussay Dellagi, Nathanael Hozé, Jaffu Chilongola, Gheyath K. Nasrallah, Simon Cauchemez, Henrik Salje

**Affiliations:** 1 Department of Genetics, University of Cambridge, Cambridge, UK; 2 Department of Epidemiology of Microbial Diseases, Yale School of Public Health, New Haven, CT USA; 3 Coalition for Epidemic Preparedness Innovations, London, UK; 4 Sokoine University of Agriculture, Morogoro, Tanzania; 5 Rwanda Military Teaching Hospital and University of Rwanda, Kigali, Rwanda; 6 York University, Toronto, ON Canada; 7 Department of Biostatistics, School of Public Health, University of Ghana, Accra, Ghana; 8 University of Ilorin, Ilorin, Nigeria; 9 Pasteur Network, Paris, France; 10 Université Paris Cité, INSERM, IAME, Paris, France; 11 Institut Pasteur, Epidemiology and Modelling of Antimicrobials Evasion Research Unit, Paris, France; 12 Université Paris-Saclay, UVSQ, INSERM, CESP, Anti-infective Evasion and Pharmacoepidemiology Research Team, Montigny-Le-Bretonneux, France; 13 Kilimanjaro Christian Medical University College, Moshi, Tanzania; 14 College of Health Sciences, Qatar University, Doha, Qatar; 15 MathematicModelling of Infectious Diseases Unit, Institut Pasteur, Université Paris Cité, UMR 2000 CNRS, Paris, France

**Keywords:** Viral infection, Epidemiology, Health policy

Correction to: *Nature Medicine* 10.1038/s41591-025-03703-w, published online 10 June 2025.

In the version of the article initially published, our code regarding the calculation of Years of Life lived with a Disability (YLDs) was incorrect, affecting our estimated numbers of Disability Adjusted Life Years (DALYs). None of the other estimates (infections, cases, deaths, doses used) were affected. The net effect is to increase the annual DALY burden from chikungunya from 121,000 to 284,000 and the number of DALYs averted per 100,000 vaccine doses used from 17 to 37. The corrections affect the DALYs estimates in the main text, in Fig. 3b and in Supplementary Tables 3 and 4 as well as estimates of averted DALYs presented in the main text, in Figs. 4, 5 and in Extended Data Fig. 3a. The HTML and PDF versions of the article have been corrected and the original, uncorrected Figs. 3–5 and Extended Data Fig. 3 are included as Supplementary information alongside this notice for comparison.

## Supplementary information

Original, uncorrected Figs. 3–5 and Extended Data Fig. 3

