# Supplementary material for: Global burden of chikungunya virus infections and the potential benefit of vaccination campaigns
Source: Nat Med. 2025 Jun 10;31(7):2342–9. doi: 10.1038/s41591-025-03703-w (PMC12283390; doi:10.1038/s41591-025-03703-w)
Supplement: Supplementary file 2 — Supplementary Table 1 [file 41591_2025_3703_MOESM2_ESM.pdf]

	Years with CHIKV detected	References
Afghanistan	-	
Albania	-	
Algeria	-	-
Angola		
Argentina	2016	[1]
Armenia	-	-
Australia	No Cases	[1]
Austria	No Cases	[1]
Azerbaijan	-	
Bahamas, The	2014<br>2015<br>2016	
Bahrain	-	
Bangladesh	<2010<br>2011<br>2012<br>2017	[2–5]
Barbados	2014<br>2015<br>2016<br>2018<br>2019<br>2020<br>2021	[6]
Belarus	-	-
Belgium	No Cases	[1]
Belize		
Benin	<2010	[7]
Bhutan	2012	[1,8]
Bolivia	2015<br>2016<br>2017<br>2018	[1,6]

	2019<br>2020<br>2021<br>2022	
Bosnia and Herzegovina	No Data	
Botswana	No Data	
Brazil	2014<br>2015<br>2016<br>2017<br>2018<br>2019<br>2020<br>2021<br>2022	1,9–16
Brunei	2011	1,17
Bulgaria	No Data	
Burkina Faso	<2010<br>2016	18,19
Burundi	<2010	20
Cambodia	<2010<br>2011<br>2012<br>2013<br>2020<br>2021	20–25
Cameroon	<2010<br>2013	19,26–29
Canada	No Cases	1
Cape Verde/ Cabo Verde	No Cases	1
Central African Republic	<2010	26,30
Chad	2020	31,32

Chile	2014<br>2022	6,33
China	<2010<br>2017	1,34,35
Colombia	2014<br>2015<br>2016<br>2017<br>2018<br>2019<br>2020<br>2021<br>2022	6,36–40
Comoros	<2010	26,41
Congo (Brazzaville)	2011<br>2019<br>2020	42,43
Congo (Kinshasa)	<2010<br>2019<br>2021	22,26,44–46
Costa Rica	2014<br>2015<br>2016<br>2017<br>2018<br>2019<br>2020<br>2021<br>2022	6,47
Croatia	No Cases	1
Cuba	2014<br>2015	6
Cyprus	No Data	
Czech Republic	No cases	1
Denmark	No cases	1

Djibouti	<2010<br>2019	[48,49]
Dominican Republic	2014<br>2015<br>2016	[1,6]
East Timor (Timor Leste)	No Data	
Ecuador	2014<br>2015<br>2016<br>2017<br>2018<br>2019<br>2020	[1,6,50–52]
Egypt	No data	
El Salvador	2014<br>2015<br>2016<br>2017<br>2018<br>2019<br>2020<br>2021	[1,6]
Equatorial Guinea	No Cases	[1]
Eritrea	No Data	
Estonia	No Cases	[1]
Ethiopia	2019<br>2022	[26]
Fiji	2015<br>2016	
Finland	No Cases	[1]
France	<2010<br>2014	[1]
French Guiana	2013<br>2014<br>2015<br>2016	[1,6,53,54]

	2017<br>2018	
French Polynesia	No Cases	1
Gabon	<2010<br>2012	55–58
Gambia	-	-
Georgia	No Cases	1
Germany	No Cases	1
Ghana	<2010	19,59
Greece	No Cases	1
Guatemala	2014<br>2015<br>2016<br>2017<br>2018<br>2019<br>2020<br>2021<br>2022	6,60,61
Guinea	<2010	26
Guinea Bissau (Guinea-Bissau)	No Data	
Guyana	2014<br>2015<br>2016	1,6
Haiti	2014<br>2016<br>2018<br>2019	6
Honduras	2013<br>2014<br>2015<br>2016<br>2018<br>2019<br>2020<br>2021	6,62

	2022	
Hungary	No Cases	1
Iceland	No Cases	1
India	<2010<br>2011<br>2012<br>2013<br>2015<br>2016<br>2017<br>2018<br>2019<br>2020<br>2021<br>2022	63
Indonesia	<2010<br>2014<br>2015<br>2016	64–68
Iran		
Iraq	No Cases	1
Ireland	No Cases	1
Israel	<2010	69
Italy	<2010<br>2016	1
Ivory Coast (Ctte d'Ivoire)	No Data	
Jamaica	2014<br>2015<br>2016<br>2018<br>2019	1,6
Japan	No local transmission	1
Jordan	No Data	
Kazakhstan	No Data	
Kenya	<2010	1,26,70–74

	2016<br>2017<br>2018<br>2019<br>2020<br>2021<br>2022	
Korea, North	No Data	
Korea, South	No Data	
Kosovo	No Data	
Kuwait	No Data	
Kyrgyzstan	No Data	
Laos	2013	[75]
Latvia	No Case	[1]
Lebanon	No Data	
Lesotho	No Data	
Liberia	<2010	[76]
Libya	No Data	
Lithuania	No Cases	[1]
Luxembourg	No Cases	[1]
Macedonia, The former Yugoslav Republic of	No Data	
Madagascar	<2010	[26,77]
Malawi	<2010	[78]
Malaysia	<2010<br>2011<br>2013<br>2020<br>2022	[66,79–83]
Maldives	<2010<br>2019	[84]
Mali	<2010	[85,86]
Malta	No Cases	[1]

Mauritania		
Mauritius	<2010	[87,88]
Mexico	2013<br>2014<br>2015<br>2016<br>2017<br>2018<br>2019<br>2020<br>2021<br>2022	[1,6,89,90]
Moldova	No Data	
Mongolia	No Cases	[1]
Montenegro	No Data	
Morocco	No Cases	[1]
Mozambique	<2010<br>2012<br>2013<br>2014<br>2015<br>2016	[26,91]
Myanmar	<2010<br>2016<br>2019	[92,93]
Namibia	<2010 (seroprevalence)	(Joubert et al. 1985)
Nepal	2013<br>2016<br>2017	[1,94]
Netherlands	No Cases	[1]
New Caledonia	No Cases	[1]
New Zealand	No Cases	[1]
Nicaragua	2014<br>2015<br>2016	[1,6,22,95]

	2017<br>2018<br>2019<br>2020<br>2021<br>2022	
Niger	<2010	
Nigeria	<2010<br>2017	[26,96,97]
Norway	No Cases	[1]
Oman	<2010	[98]
Pakistan	2017	ECDC
Panama	2015<br>2016<br>2017<br>2018<br>2019<br>2022	[6,99–101]
Papua New Guinea	2012	[1,102]
Paraguay	2014<br>2015<br>2016<br>2017<br>2018<br>2019<br>2020<br>2021<br>2022	[6,103,104]
Peru	2014<br>2015<br>2016<br>2017<br>2018<br>2019<br>2020<br>2021<br>2022	[6,105–107]
Philippines	<2010	[108–110]

	2011<br>2012<br>2014<br>2022	
Poland	No Cases	1
Portugal	No Cases	1
Puerto Rico	2014<br>2015<br>2016<br>2017	6,111
Qatar	No Cases	
Romania	No Cases	1
Russia	No Cases	1
Rwanda	No Data	
Samoa	No Cases	1
Sao Tome and Principe	No Data	
Saudi Arabia	2019	112
Senegal	<2010<br>2015	26,113,114
Serbia	No Data	
Sierra Leone	<2010<br>2012<br>2013	26,115
Singapore	<2010<br>2013<br>2019	116–119
Slovakia	No Cases	1
Slovenia	No Cases	1
Solomon Islands	No Data	
Somalia	2016	26
Somaliland	No Data	
South Africa	<2010	120
South Sudan	No Data	

Spain	No Data	
Sri Lanka	<2010	68,121,122
Sudan	<2010<br>2018<br>2019<br>2020<br>2022	1,26,55,123
Suriname	2014<br>2015<br>2018<br>2019<br>2022	6,124,125
Swaziland/ Eswatini	No Data	
Sweden	No Cases	1
Switzerland	No Cases	1
Syria	No Data	
Taiwan	2019	126
Tajikistan	No Data	
Tanzania	<2010<br>2013<br>2014<br>2015<br>2016<br>2018	1,19,26,127,128
Thailand	<2010<br>2018<br>2019<br>2020<br>2022	1,129,130
Togo	No Data	
Trinidad and Tobago	2014	1
Tunisia	No Data	
Turkey		
Turkmenistan	No Data	
Uganda	<2010	26,131,132

Ukraine	No Data	
United Arab Emirates	No Data	
United Kingdom	No Cases	1
United States of America	2014<br>2018<br>2022	6<br>1
Uruguay	2014<br>2022	6,133
Uzbekistan	No Data	
Vanuatu	No Cases	1
Venezuela	2014<br>2015<br>2016<br>2017<br>2018<br>2019<br>2020<br>2021<br>2022	6,134–136
Vietnam	<2010	68<br>137
Western Sahara	No Data	
Yemen	<2010<br>2011<br>2012<br>2020	138,139
Zambia	<2010<br>2016	19,26,140
Zimbabwe	<2010	19,26

References

1. Inc Gideon Informatics & Berger, S. *Chikungunya and Zika Global Status*. (Gideon

Informatics, Incorporated, 2021).

2. Khatun, S. *et al.* An Outbreak of Chikungunya in Rural Bangladesh, 2011. *PLoS Negl. Trop. Dis.* **9**, e0003907 (2015).
3. Icddr, B. First identified outbreak of chikungunya in Bangladesh, 2008. *Health Sci Bull.*
4. Salje, H. *et al.* How social structures, space, and behaviors shape the spread of infectious diseases using chikungunya as a case study. *Proc. Natl. Acad. Sci. U. S. A.* **113**, 13420–13425 (2016).
5. Kabir, I., Dhimal, M., Müller, R., Banik, S. & Haque, U. The 2017 Dhaka chikungunya outbreak. *Lancet Infect. Dis.* **17**, 1118 (2017).
6. Gutiérrez, L. A. PAHO/WHO Data - Weekly Report. *Pan American Health Organization / World Health Organization* https://www3.paho.org/data/index.php/en/mnu-topics/chikv-en/550-chikv-weekly-en.html (2019).
7. Bacci, A. *et al.* High seroprevalence of chikungunya virus antibodies among pregnant women living in an urban area in Benin, West Africa. *Am. J. Trop. Med. Hyg.* **92**, 1133–1136 (2015).
8. Wangchuk, S. *et al.* Chikungunya fever outbreak, Bhutan, 2012. *Emerg. Infect. Dis.* **19**, 1681–1684 (2013).
9. TabNet Win32 3.0: Febre de Chikungunya - Notificações registradas no Sistema de Informação de Agravos de Notificação - São Paulo. http://tabnet.datasus.gov.br/cgi/tabcgi.exe?sinannet/cnv/chikunsp.def.
10. Silva, M. M. O. *et al.* Concomitant Transmission of Dengue, Chikungunya, and Zika Viruses in Brazil: Clinical and Epidemiological Findings From Surveillance for Acute Febrile Illness. *Clin. Infect. Dis.* **69**, 1353–1359 (2019).
11. Simião, A. R. *et al.* A major chikungunya epidemic with high mortality in northeastern Brazil. *Rev. Soc. Bras. Med. Trop.* **52**, e20190266 (2019).
12. Dias, J. P. *et al.* Seroprevalence of Chikungunya Virus after Its Emergence in Brazil. *Emerg. Infect. Dis.* **24**, 617–624 (2018).
13. Silva, N. M. da *et al.* Chikungunya surveillance in Brazil: challenges in the context of

Public Health. *Epidemiol Serv Saude* **27**, e2017127 (2018).

14. Silva Junior, G. B. da, Pinto, J. R., Mota, R. M. S., Pires Neto, R. da J. & Daher, E. D. F. Risk factors for death among patients with Chikungunya virus infection during the outbreak in northeast Brazil, 2016-2017. *Trans. R. Soc. Trop. Med. Hyg.* **113**, 221–226 (2019).
15. Naveca, F. G. *et al.* Genomic, epidemiological and digital surveillance of Chikungunya virus in the Brazilian Amazon. *PLoS Negl. Trop. Dis.* **13**, e0007065 (2019).
16. Albuquerque, I. G. C. de *et al.* Chikungunya virus infection: report of the first case diagnosed in Rio de Janeiro, Brazil. *Rev. Soc. Bras. Med. Trop.* **45**, 128–129 (2012).
17. Liew, C. & Yung, C. F. First detection of chikungunya infection and transmission in Brunei Darussalam. *Singapore Med. J.* **53**, e66–8 (2012).
18. Hien, A. S. *et al.* Chikungunya (Togaviridae) and Dengue 2 (Flaviviridae) Viruses Isolated from Aedes aegypti Mosquitoes by qRT-PCR Technique: Xenosurveillance for Arboviruses Circulating in Burkina Faso. *Preprints* (2021) doi:10.20944/preprints202108.0077.v1.
19. Eisenhut, M., Schwarz, T. F. & Hegenscheid, B. Seroprevalence of dengue, chikungunya and Sindbis virus infections in German aid workers. *Infection* **27**, 82–85 (1999).
20. Rodhain, F., Carteron, B., Laroche, R. & Hannoun, C. [Human arbovirus infections in Burundi: results of a seroepidemiologic survey, 1980-1982]. *Bull. Soc. Pathol. Exot. Filiales* **80**, 155–161 (1987).
21. Ministry of Health warns of rising Chikungunya cases in the Kingdom - Khmer Times. *Khmer Times - Insight into Cambodia* https://www.khmertimeskh.com/50874184/ministry-of-health-warns-of-rising-chikungunya-cases-in-the-kingdom/ (2021).
22. Chikungunya worldwide overview. *European Centre for Disease Prevention and Control* https://www.ecdc.europa.eu/en/chikungunya-monthly (2023).
23. Centers for Disease Control and Prevention (CDC). Chikungunya outbreak--Cambodia,

February-March 2012. *MMWR Morb. Mortal. Wkly. Rep.* **61**, 737–740 (2012).

24. Sony, O. Chikungunya spreads to 21 provinces, almost 6,000 suspected infected. *VOD English* https://vodenglish.news/chikungunya-spreads-to-21-provinces-almost-6000-suspected-infected/ (2020).
25. Auerswald, H. *et al.* Broad and long-lasting immune protection against various Chikungunya genotypes demonstrated by participants in a cross-sectional study in a Cambodian rural community. *Emerg. Microbes Infect.* **7**, 13 (2018).
26. Russo, G., Subissi, L. & Rezza, G. Chikungunya fever in Africa: a systematic review. *Pathog. Glob. Health* **114**, 136–144 (2020).
27. Maurice, D. *et al.* Molecular characterization of chikungunya virus from three regions of Cameroon. *Virol. Sin.* **30**, 470–473 (2015).
28. Demanou, M. *et al.* Chikungunya outbreak in a rural area of Western Cameroon in 2006: A retrospective serological and entomological survey. *BMC Res. Notes* **3**, 128 (2010).
29. Kuniholm, M. H. *et al.* Seroprevalence and distribution of Flaviviridae, Togaviridae, and Bunyaviridae arboviral infections in rural Cameroonian adults. *Am. J. Trop. Med. Hyg.* **74**, 1078–1083 (2006).
30. Sureau, P., Jaeger, G., Pinerd, G., Palisson, M. J. & Bedaya-N’Garo, S. [Sero-epidemiological survey of arbovirus diseases in the Bi-Aka pygmies of Lobaye, Central African Republic]. *Bull. Soc. Pathol. Exot. Filiales* **70**, 131–137 (1977).
31. Chikungunya – Chad. https://www.who.int/emergencies/disease-outbreak-news/item/chikungunya-chad.
32. Yonga, M. G. *et al.* Molecular characterization of chikungunya virus from the first cluster of patients during the 2020 outbreak in Chad. *Arch. Virol.* **167**, 1301–1305 (2022).
33. Perret, C. *et al.* Chikungunya, emerging disease in Latin America. Description of the first cases in Chile. *Rev. Chilena Infectol.* **35**, 413–419 (2018).
34. Zheng, K. *et al.* Genetic analysis of chikungunya viruses imported to mainland China in 2008. *Virol. J.* **7**, 8 (2010).

35. Wu, D. *et al.* Chikungunya outbreak in Guangdong Province, China, 2010. *Emerg. Infect. Dis.* **18**, 493–495 (2012).

36. Rueda, J. C. *et al.* Demographic and clinical characteristics of chikungunya patients from six Colombian cities, 2014-2015. *Emerg. Microbes Infect.* **8**, 1490–1500 (2019).

37. Rodas, J. D. *et al.* Genetic Characterization of Northwestern Colombian Chikungunya Virus Strains from the 2014-2015 Epidemic. *Am. J. Trop. Med. Hyg.* **95**, 639–646 (2016).

38. Oviedo-Pastrana, M., Méndez, N., Mattar, S., Arrieta, G. & Gomezcaceres, L. Lessons learned of emerging Chikungunya virus in two populations of social vulnerability of the Colombian tropics: epidemiological analysis. *Arch. Public Health* **76**, 36 (2018).

39. Vidal, O. M. *et al.* Chikungunya outbreak (2015) in the Colombian Caribbean: Latent classes and gender differences in virus infection. *PLoS Negl. Trop. Dis.* **14**, e0008281 (2020).

40. Pacheco, Ó. *et al.* [Estimation of underreporting of Chikungunya virus infection cases in Girardot, Colombia, from November, 2014, to May, 2015]. *Biomedica* **37**, 507–515 (2017).

41. Sergon, K. *et al.* Seroprevalence of Chikungunya virus infection on Grande Comore Island, union of the Comoros, 2005. *Am. J. Trop. Med. Hyg.* **76**, 1189–1193 (2007).

42. Mombouli, J.-V. *et al.* Chikungunya virus infection, Brazzaville, Republic of Congo, 2011. *Emerg. Infect. Dis.* **19**, 1542–1543 (2013).

43. Fritz, M. *et al.* Re-emergence of chikungunya in the Republic of the Congo in 2019 associated with a possible vector-host switch. *Int. J. Infect. Dis.* **84**, 99–101 (2019).

44. Pastorino, B. *et al.* Epidemic resurgence of Chikungunya virus in democratic Republic of the Congo: identification of a new central African strain. *J. Med. Virol.* **74**, 277–282 (2004).

45. Muyembe-Tamfum, J. J. *et al.* [Epidemic of Chikungunya virus in 1999 and 200 in the Democratic Republic of the Congo]. *Med. Trop.* **63**, 637–638 (2003).

46. Selhorst, P. *et al.* Molecular characterization of chikungunya virus during the 2019

outbreak in the Democratic Republic of the Congo. *Emerg. Microbes Infect.* **9**, 1912–1918 (2020).

47. Luksic, B. *et al.* First case of imported chikungunya infection in Croatia, 2016. *Int Med Case Rep J* **10**, 117–121 (2017).
48. Fourié, T. *et al.* Emergence of Indian lineage of ECSA chikungunya virus in Djibouti, 2019. *Int. J. Infect. Dis.* **108**, 198–201 (2021).
49. Andayi, F. *et al.* A sero-epidemiological study of arboviral fevers in Djibouti, Horn of Africa. *PLoS Negl. Trop. Dis.* **8**, e3299 (2014).
50. Chis Ster, I. *et al.* Age-dependent seroprevalence of dengue and chikungunya: inference from a cross-sectional analysis in Esmeraldas Province in coastal Ecuador. *BMJ Open* **10**, e040735 (2020).
51. Cevallos, V. *et al.* Zika and Chikungunya virus detection in naturally infected Aedes aegypti in Ecuador. *Acta Trop.* **177**, 74–80 (2018).
52. Quillupangui, S. Tres mil nuevos contagios de chikungunya en Ecuador. *El Comercio* https://www.elcomercio.com/tendencias/ecuador-chikungunya-enfermedades-contagios-mosquitos.html.
53. Bailly, S. *et al.* Spatial Distribution and Burden of Emerging Arboviruses in French Guiana. *Viruses* **13**, (2021).
54. Bonifay, T. *et al.* Atypical and severe manifestations of chikungunya virus infection in French Guiana: A hospital-based study. *PLoS One* **13**, e0207406 (2018).
55. Bettis, A. A. *et al.* The global epidemiology of chikungunya from 1999 to 2020: A systematic literature review to inform the development and introduction of vaccines. *PLoS Negl. Trop. Dis.* **16**, e0010069 (2022).
56. Nkoghe, D. *et al.* Clinical forms of chikungunya in Gabon, 2010. *PLoS Negl. Trop. Dis.* **6**, e1517 (2012).
57. Home - ProMED. *ProMED-mail* https://promedmail.org/promed-post/?id=20121219.1458587 (2019).
58. Gabor, J. J., Schwarz, N. G., Esen, M., Kremsner, P. G. & Grobusch, M. P. Dengue

and chikungunya seroprevalence in Gabonese infants prior to major outbreaks in 2007 and 2010: A sero-epidemiological study. *Travel Med. Infect. Dis.* **14**, 26–31 (2016).

59. Adusei, J. A. *et al.* Evidence of chikungunya virus infections among febrile patients at three secondary health facilities in the Ashanti and the Bono Regions of Ghana. *PLoS Negl. Trop. Dis.* **15**, e0009735 (2021).
60. Carrillo-Soto, M. A. & Montoya-Valladares, A. S. A propósito de 3 casos con retinopatía posterior a fase aguda de fiebre por chikungunya. *Archivos de la Sociedad Española de Oftalmología* **95**, 408–410 (2020).
61. Edwards, T. *et al.* Co-infections with Chikungunya and Dengue Viruses, Guatemala, 2015. *Emerg. Infect. Dis.* **22**, 2003–2005 (2016).
62. Ortiz-Quezada, J. *et al.* Chikungunya encephalitis, a case series from an endemic country. *J. Neurol. Sci.* **420**, 117279 (2021).
63. Ministry of Health & Family Welfare-Government of India. Chikungunya situation in India. https://ncvbdc.mohfw.gov.in/index4.php?lang=1&level=0&linkid=486&lid=3765.
64. Thousands contract chikungunya in Lampung. https://wildsingaporenews.blogspot.com/2010/01/thousands-contract-chikungunya-in.html.
65. Harapan, H. *et al.* Chikungunya virus infection in Indonesia: a systematic review and evolutionary analysis. *BMC Infect. Dis.* **19**, 243 (2019).
66. Tesh, R. B., Gajdusek, D. C., Garruto, R. M., Cross, J. H. & Rosen, L. The distribution and prevalence of group A arbovirus neutralizing antibodies among human populations in Southeast Asia and the Pacific islands. *Am. J. Trop. Med. Hyg.* **24**, 664–675 (1975).
67. Porter, K. R. *et al.* A serological study of Chikungunya virus transmission in Yogyakarta, Indonesia: evidence for the first outbreak since 1982. *Southeast Asian J. Trop. Med. Public Health* **35**, 408–415 (2004).
68. Ngwe Tun, M. M. *et al.* Retrospective seroepidemiological study of chikungunya infection in South Asia, Southeast Asia and the Pacific region. *Epidemiol. Infect.* **144**, 2268–2275 (2016).

69. Tanay, A. *et al.* Chikungunya fever in Israeli travelers returning from northwestern India. *J. Travel Med.* **15**, 382–384 (2008).

70. Maljkovic Berry, I. *et al.* Global Outbreaks and Origins of a Chikungunya Virus Variant Carrying Mutations Which May Increase Fitness for Aedes aegypti: Revelations from the 2016 Mandera, Kenya Outbreak. *Am. J. Trop. Med. Hyg.* **100**, 1249–1257 (2019).

71. Grossi-Soyster, E. N. *et al.* Serological and spatial analysis of alphavirus and flavivirus prevalence and risk factors in a rural community in western Kenya. *PLoS Negl. Trop. Dis.* **11**, e0005998 (2017).

72. Home - ProMED. *ProMED-mail* https://promedmail.org/promed-post/?id=20180420.5758774 (2019).

73. Eyase, F. *et al.* Emergence of a novel chikungunya virus strain bearing the E1:V80A substitution, out of the Mombasa, Kenya 2017-2018 outbreak. *PLoS One* **15**, e0241754 (2020).

74. News Desk. Kenya reports chikungunya outbreak. *Outbreak News Today* http://outbreaknewstoday.com/kenya-reports-chikungunya-outbreak-37499/ (2020).

75. Phommanivong, V. *et al.* Co-circulation of the dengue with chikungunya virus during the 2013 outbreak in the southern part of Lao PDR. *Trop. Med. Health* **44**, 24 (2016).

76. Woodruff, A. W., Bowen, E. T. & Platt, G. S. Viral infections in travellers from tropical Africa. *Br. Med. J.* **1**, 956–958 (1978).

77. Schwarz, N. G. *et al.* Seroprevalence of antibodies against Chikungunya, Dengue, and Rift Valley fever viruses after febrile illness outbreak, Madagascar. *Emerg. Infect. Dis.* **18**, 1780–1786 (2012).

78. van den Bosch, C. & Lloyd, G. Chikungunya fever as a risk factor for endemic Burkitt's lymphoma in Malawi. *Trans. R. Soc. Trop. Med. Hyg.* **94**, 704–705 (2000).

79. Azami, N. A. M. *et al.* Emergence of chikungunya seropositivity in healthy Malaysian adults residing in outbreak-free locations: chikungunya seroprevalence results from the Malaysian Cohort. *BMC Infect. Dis.* **13**, 67 (2013).

80. Home - ProMED. *ProMED-mail* https://promedmail.org/promed-

post/?id=20120212.1040110 (2019).

81. Kumarasamy, V. *et al.* Re-emergence of Chikungunya virus in Malaysia. *Med. J. Malaysia* **61**, 221–225 (2006).

82. Ayu, S. M. *et al.* Seroprevalence survey of Chikungunya virus in Bagan Panchor, Malaysia. *Am. J. Trop. Med. Hyg.* **83**, 1245–1248 (2010).

83. Capeding, M. R. *et al.* Dengue and other common causes of acute febrile illness in Asia: an active surveillance study in children. *PLoS Negl. Trop. Dis.* **7**, e2331 (2013).

84. Dudouet, P. *et al.* Chikungunya resurgence in the Maldives and risk for importation via tourists to Europe in 2019-2020: A GeoSentinel case series. *Travel Med. Infect. Dis.* **36**, 101814 (2020).

85. Delynn M, M. *et al.* Serological evidence of dengue and Chikungunya exposures in Malian children by multiplex bead assay. *Int J Trop Dis* **1**, (2018).

86. Safronetz, D. *et al.* Vectorborne Infections, Mali. *Emerg. Infect. Dis.* **22**, 340–342 (2016).

87. Edwards, C. J. *et al.* Molecular diagnosis and analysis of Chikungunya virus. *J. Clin. Virol.* **39**, 271–275 (2007).

88. Beesoon, S., Funkhouser, E., Kotea, N., Spielman, A. & Robich, R. M. Chikungunya fever, Mauritius, 2006. *Emerg. Infect. Dis.* **14**, 337–338 (2008).

89. Nunez-Avellaneda, D. *et al.* Chikungunya in Guerrero, Mexico, 2019 and Evidence of Gross Underreporting in the Region. *Am. J. Trop. Med. Hyg.* **105**, 1281–1284 (2021).

90. Kautz, T. F. *et al.* Chikungunya Virus as Cause of Febrile Illness Outbreak, Chiapas, Mexico, 2014. *Emerg. Infect. Dis.* **21**, 2070–2073 (2015).

91. Gudo, E. S., Falk, K. & Cliff, J. Historical Perspective of Arboviruses in Mozambique and Its Implication for Current and Future Epidemics. in *Dengue and Zika: Control and Antiviral Treatment Strategies* (eds. Hilgenfeld, R. & Vasudevan, S. G.) 11–18 (Springer Singapore, Singapore, 2018).

92. Wimalasiri-Yapa, B. M. C. R. *et al.* Chikungunya virus in Asia - Pacific: a systematic review. *Emerg. Microbes Infect.* **8**, 70–79 (2019).

93. Luvai, E. A. C. *et al.* Evidence of Chikungunya virus seroprevalence in Myanmar among dengue-suspected patients and healthy volunteers in 2013, 2015, and 2018. *PLoS Negl. Trop. Dis.* **15**, e0009961 (2021).
94. Pun, S. B., Bastola, A. & Shah, R. First report of Chikungunya virus infection in Nepal. *J. Infect. Dev. Ctries.* **8**, 790–792 (2014).
95. Communicable disease threats report, 14-20 June 2020, week 25. *European Centre for Disease Prevention and Control* https://www.ecdc.europa.eu/en/publications-data/communicable-disease-threats-report-14-20-june-2020-week-25 (2020).
96. Kolawole, O. M., Bello, K. E., Seriki, A. A. & Irekeola, A. A. Serological survey of Chikungunya virus in Ilorin Metropolis, Nigeria. *Braz. J. Infect. Dis.* **21**, 365–366 (2017).
97. Baba, M. *et al.* Evidence of arbovirus co-infection in suspected febrile malaria and typhoid patients in Nigeria. *J. Infect. Dev. Ctries.* **7**, 51–59 (2013).
98. Al-Abri, S. S. *et al.* Epidemiology of travel-associated infections in Oman 1999-2013: A retrospective analysis. *Travel Med. Infect. Dis.* **13**, 388–393 (2015).
99. Díaz, Y. *et al.* Chikungunya virus infection: first detection of imported and autochthonous cases in Panama. *Am. J. Trop. Med. Hyg.* **92**, 482–485 (2015).
100. Araúz, D. *et al.* Febrile or Exanthematous Illness Associated with Zika, Dengue, and Chikungunya Viruses, Panama. *Emerg. Infect. Dis.* **22**, 1515–1517 (2016).
101. Carrera, J.-P. *et al.* Endemic and Epidemic Human Alphavirus Infections in Eastern Panama: An Analysis of Population-Based Cross-Sectional Surveys. *Am. J. Trop. Med. Hyg.* **103**, 2429–2437 (2020).
102. Horwood, P. F. *et al.* Outbreak of chikungunya virus infection, Vanimo, Papua New Guinea. *Emerg. Infect. Dis.* **19**, 1535–1538 (2013).
103. Bogado, R. R. Reportan caso sospechoso de chikungunya en Luque. *Última Hora* https://www.ultimahora.com/reportan-caso-sospechoso-chikungunya-luque-n809010.html (2014).
104. Gräf, T. *et al.* Epidemiologic History and Genetic Diversity Origins of Chikungunya and Dengue Viruses, Paraguay. *Emerg. Infect. Dis.* **27**, 1393–1404 (2021).

105. Del Valle-Mendoza, J. *et al.* Unidentified dengue serotypes in DENV positive samples and detection of other pathogens responsible for an acute febrile illness outbreak 2016 in Cajamarca, Peru. *BMC Res. Notes* **13**, 467 (2020).

106. Alva-Urcia, C. *et al.* Emerging and reemerging arboviruses: A new threat in Eastern Peru. *PLoS One* **12**, e0187897 (2017).

107. Sánchez-Carbonel, J. *et al.* Identification of infection by Chikungunya, Zika, and Dengue in an area of the Peruvian coast. Molecular diagnosis and clinical characteristics. *BMC Res. Notes* **11**, 175 (2018).

108. Montemayor, M. T. Chikungunya cases 169% higher than 2021. *Philippine News Agency* https://www.pna.gov.ph/articles/1176376 (2022).

109. Yoon, I.-K. *et al.* High rate of subclinical chikungunya virus infection and association of neutralizing antibody with protection in a prospective cohort in the Philippines. *PLoS Negl. Trop. Dis.* **9**, e0003764 (2015).

110. Srikiatkhachorn, A. *et al.* Resolution of a Chikungunya Outbreak in a Prospective Cohort, Cebu, Philippines, 2012-2014. *Emerg. Infect. Dis.* **22**, 1852–1854 (2016).

111. Freitas, A. R. R., Donalisio, M. R. & Alarcón-Elbal, P. M. Excess Mortality and Causes Associated with Chikungunya, Puerto Rico, 2014-2015. *Emerg. Infect. Dis.* **24**, 2352–2355 (2018).

112. Hakami, A. R. *et al.* Detection of chikungunya virus in the Southern region, Saudi Arabia. *Virol. J.* **18**, 190 (2021).

113. Chikungunya – Senegal. https://www.who.int/emergencies/disease-outbreak-news/item/14-september-2015-chikungunya-en.

114. Sow, A. *et al.* Chikungunya Outbreak in Kedougou, Southeastern Senegal in 2009-2010. *Open Forum Infect Dis* **5**, ofx259 (2018).

115. Ansumana, R. *et al.* Reemergence of chikungunya virus in Bo, Sierra Leone. *Emerg. Infect. Dis.* **19**, 1108–1110 (2013).

116. Migration. Spike in number of chikungunya cases in Singapore. *The Straits Times* https://www.straitstimes.com/singapore/spike-in-number-of-chikungunya-cases-in-

singapore (2013).

117. Ang, L. W. *et al.* Seroprevalence of antibodies against chikungunya virus in Singapore resident adult population. *PLoS Negl. Trop. Dis.* **11**, e0006163 (2017).

118. Leo, Y. S. *et al.* Chikungunya outbreak, Singapore, 2008. *Emerg. Infect. Dis.* **15**, 836–837 (2009).

119. Auto, H. Singapore students contract chikungunya fever on service learning trip to Thailand. *Rei Kurohi* https://www.straitstimes.com/singapore/health/singapore-students-contract-chikungunya-fever-on-service-learning-trip-to-thailand (2019).

120. Fourie, E. D. & Morrison, J. G. Rheumatoid arthritic syndrome after chikungunya fever. *S. Afr. Med. J.* **56**, 130–132 (1979).

121. Kularatne, S. A. M. *et al.* Epidemiology, clinical manifestations, and long-term outcomes of a major outbreak of chikungunya in a hamlet in sri lanka, in 2007: a longitudinal cohort study. *J. Trop. Med.* **2012**, 639178 (2012).

122. Mohanty, I. *et al.* Seroprevalence of chikungunya in southern odisha. *J Family Med Prim Care* **2**, 33–36 (2013).

123. Farnon, E. C. *et al.* Household-based sero-epidemiologic survey after a yellow fever epidemic, Sudan, 2005. *Am. J. Trop. Med. Hyg.* **82**, 1146–1152 (2010).

124. van Genderen, F. T. *et al.* First Chikungunya Outbreak in Suriname; Clinical and Epidemiological Features. *PLoS Negl. Trop. Dis.* **10**, e0004625 (2016).

125. Goeijenbier, M. *et al.* Emerging Viruses in the Republic of Suriname: Retrospective and Prospective Study into Chikungunya Circulation and Suspicion of Human Hantavirus Infections, 2008-2012 and 2014. *Vector Borne Zoonotic Dis.* **15**, 611–618 (2015).

126. Chen, M.-Y. *et al.* Chikungunya infection: First autochthonous cases in Taiwan. *J. Formos. Med. Assoc.* **120**, 1526–1530 (2021).

127. Kajeguka, D. C. *et al.* Prevalence of dengue and chikungunya virus infections in north-eastern Tanzania: a cross sectional study among participants presenting with malaria-like symptoms. *BMC Infect. Dis.* **16**, 183 (2016).

128. Chipwaza, B. *et al.* Occurrence of 4 Dengue Virus Serotypes and Chikungunya Virus in

Kilombero Valley, Tanzania, During the Dengue Outbreak in 2018. *Open Forum Infect Dis* **8**, ofaa626 (2021).

129. Imad, H. A. *et al.* Chikungunya Manifestations and Viremia in Patients WhoPresented to the Fever Clinic at Bangkok Hospital for Tropical Diseases during the 2019 Outbreak in Thailand. *Trop Med Infect Dis* **6**, (2021).
130. Laoprasopwattana, K., Suntharasaj, T., Petmanee, P., Suddeaugrai, O. & Geater, A. Chikungunya and dengue virus infections during pregnancy: seroprevalence, seroincidence and maternal-fetal transmission, southern Thailand, 2009-2010. *Epidemiol. Infect.* **144**, 381–388 (2016).
131. Clements, T. L. *et al.* Chikungunya and O'nyong-nyong Viruses in Uganda: Implications for Diagnostics. *Open Forum Infect Dis* **6**, ofz001 (2019).
132. Rodhain, F. *et al.* Arbovirus infections and viral haemorrhagic fevers in Uganda: a serological survey in Karamoja district, 1984. *Trans. R. Soc. Trop. Med. Hyg.* **83**, 851–854 (1989).
133. GIDEON platform. https://app.gideononline.com/references/m/20140823.2717133.
134. Carrillo-Hernández, M. Y., Ruiz-Saenz, J., Villamizar, L. J., Gómez-Rangel, S. Y. & Martínez-Gutierrez, M. Co-circulation and simultaneous co-infection of dengue, chikungunya, and zika viruses in patients with febrile syndrome at the Colombian-Venezuelan border. *BMC Infect. Dis.* **18**, 61 (2018).
135. Torres, J. R. *et al.* Chikungunya fever: Atypical and lethal cases in the Western hemisphere: A Venezuelan experience. *IDCases* **2**, 6–10 (2015).
136. Lizarazo, E. *et al.* Spatial Dynamics of Chikungunya Virus, Venezuela, 2014. *Emerg. Infect. Dis.* **25**, 672–680 (2019).
137. Powers, A. M. & Logue, C. H. Changing patterns of chikungunya virus: re-emergence of a zoonotic arbovirus. *J. Gen. Virol.* **88**, 2363–2377 (2007).
138. Malik, M. R. *et al.* Chikungunya outbreak in Al-Hudaydah, Yemen, 2011: epidemiological characterization and key lessons learned for early detection and control. *J. Epidemiol. Glob. Health* **4**, 203–211 (2014).

139. Rezza, G. *et al.* Co-circulation of Dengue and Chikungunya Viruses, Al Hudaydah, Yemen, 2012. *Emerg. Infect. Dis.* **20**, 1351–1354 (2014).

140. Chisenga, C. C. *et al.* Sero-prevalence of arthropod-borne viral infections among Lukanga swamp residents in Zambia. *PLoS One* **15**, e0235322 (2020).
