# Supplementary material for: Global burden of chikungunya virus infections and the potential benefit of vaccination campaigns
Source: Nat Med. 2025 Jun 10;31(7):2342–9. doi: 10.1038/s41591-025-03703-w (PMC12283390; doi:10.1038/s41591-025-03703-w)
Supplement: Supplementary file 3 — Supplementary Table 2 [file 41591_2025_3703_MOESM3_ESM.pdf]

A

Criteria	Inclusion	Exclusion
**Study type**	Original cross-sectional seroprevalence studies	Literature review, meta-analysis, clinical report
**Target population**	General population, healthy pregnant individuals	Symptomatic patients, suspected cases
**Target antibody**	Measuring anti-CHIKV IgG antibody titers	Measuring IgA or IgM combined with IgG antibodies titers
**Target pathogen**	CHIKV	Any collapsing of results between CHIKV and other arboviruses or related pathogens
**Target age**	Any	No exclusion criteria related to age
**Data aggregation**	Any level of aggregation across age groups was included - spatially aggregated results were included if within the country	Spatially aggregated results across different countries

B

Country	Subnational area	Year	Target population	Publically available	Link of article	Title of article	Responsible co-author
Bangladesh	Throughout	2015	general population	Yes	https://academic.oup.com/jid/article/230/5/e1031/7701446?login=true	Results of a Nationally Representative Seroprevalence Survey of Chikungunya Virus in Bangladesh	
Benin	Cotonou	2006	Pregnant women	Yes	https://www.ncbi.nlm.nih.gov/pmc/articles/PMC4458815/	High Seroprevalence of Chikungunya Virus Antibodies Among Pregnant Women Living in an Urban Area in Benin, West Africa	-
Brazil	Cruzeiro do Sul, Alto Jurua	2015-2016	women in third trimesters of pregnancy	Yes	https://www.scielo.br/j/rimtsp/a/5nFxV3sgKT3XFwqN9dqjCmK/?lang=en#	Silent circulation of Chikungunya virus among pregnant women and newborns in the Western Brazilian Amazon before the first outbreak of chikungunya fever	-
Brazil	Pernambuco , Bahia	2017	general population	Yes	https://journals.plos.org/plosntds/article?id=10.1371/journal.pntd.0009468	Anti-chikungunya virus seroprevalence in Indigenous groups in the São Francisco Valley, Brazil	-
Brazil	Salvador	2016-2017	general population	Yes	https://wwwnc.cdc.gov/eid/article/26/7/19-0846_article	Transmission of Chikungunya Virus in an Urban Slum, Brazil	-
Brazil	Juazeiro do Norte	2018	general population	Yes	https://link.springer.com/article/10.1186/s12879-020-05611-5	Seroprevalence, spatial dispersion and factors associated with flavivirus and chikungunya infection in a risk area: a population-based seroprevalence study in Brazil	-
Brazil	Quixada	2019	general population	Yes	https://onlinelibrary.wiley.com/doi/full/10.1111/zph.12888	Seroepidemiological survey on chikungunya in endemic zones for arboviruses in Brazil, 2019	-
Brazil	Chapada	2016-20	General cohort	Yes	https://journals.plos.org/plosntds/article?id=10.1371/journal.pntd.0005319	Seroprevalence of Chikungunya Virus in a Rural Community in Brazil	-
Brazil	Distrito Federal	2018	Blood donors	Yes	https://onlinelibrary.wiley.com/doi/full/10.1111/tme.12870	Chikungunya virus seroprevalence in asymptomatic blood donors during an outbreak in the Federal District of Brazil	-
Brazil	Rio de Janeiro	2018	General population	Yes	https://journals.plos.org/plosone/article?id=10.1371/journal.pone.0243239	Zika, dengue and chikungunya population prevalence in Rio de Janeiro city, Brazil, and the importance of seroprevalence studies to estimate the real number of infected individuals	-

Brazil	Ribeirao Preto & Macapa	2015-16	Blood donors	Yes	https://www.sciencedirect.com/science/article/pii/S2531137918300841	Seroprevalence of Chikungunya virus in blood donors from Northern and Southeastern Brazil	-
Burkina Faso	Ouagadougou	2015	general population	Yes	https://academic.oup.com/jid/article/227/2/261/6609553	Seroepidemiological Reconstruction of Long-term Chikungunya Virus Circulation in Burkina Faso and Gabon	-
Cambodia	Trapeang Roka village	2016	general population	Yes	https://www.ncbi.nlm.nih.gov/pmc/articles/PMC5837154/	Broad and long-lasting immune protection against various Chikungunya genotypes demonstrated by participants in a cross-sectional study in a Cambodian rural community	-
Cameroon	Kumbo	2007	healthy adults	Yes	https://pubmed.ncbi.nlm.nih.gov/20444282/	Chikungunya outbreak in a rural area of Western Cameroon in 2006: A retrospective serological and entomological survey	-
Cameroon	9 villages	2006	Healthy adults (>16yo)	Yes	https://www.ajtmh.org/view/journals/tpmd/74/6/article-p1078.xml	Seroprevalence and Distrubution of Flaviviridae, Togaviridae, and Bunyaviridae Arboviral Infections in Rural Cameroonian Adults	-
CAR	Bagandou	1975	general population	Yes	https://gallica.bnf.fr/ark:/12148/bpt6k9822827b/f36.item		-
Comoros	throughout	2015	general population	No	https://www.ncbi.nlm.nih.gov/pmc/articles/PMC5157944/pdf/pntd.0004840.pdf	Serological Evidence of Contrasted Exposure to Arboviral Infections between Islands of theUnion of Comoros (Indian Ocean)	Koussay Dellagi
Comoros	Grande Comore Island	2005	general population	Yes	https://www.ajtmh.org/view/journals/tpmd/76/6/article-p1189.xml	Seroprevalence of Chikungunya Virus Infection on Grande Comore Island, Union of the Comoros, 2005.	-
Djibouti	Djibouti city	2010-11	general population	Yes	https://www.ncbi.nlm.nih.gov/pmc/articles/PMC4263616/	A Sero-epidemiological Study of Arboviral Fevers in Djibouti, Horn of Africa	-
Ecuador	Quininde	2016	general population	Yes	https://bmjopen.bmj.com/content/10/10/e040735.abstract	Age-dependent seroprevalence of dengue and chikungunya: inference from a cross-sectional analysis in Esmeraldas Province in coastal Ecuador	-
Ethiopia	Gambella region (Lare and Itang)	2019	general population	Yes	Seroprevalence of Yellow fever, Chikungunya, and Zika virus at a community level in the Gambella Region, South West Ethiopia	Seroprevalence of Yellow fever, Chikungunya, and Zika virus at a community level in the Gambella Region, South West Ethiopia	-

Ethiopia	South Omo Valley	2018	general population	Yes	https://journals.plos.org/plosntds/article?id=10.1371/journal.pntd.0008549	Community-based sero-prevalence of chikungunya and yellow fever in the South Omo Valley of Southern Ethiopia	-
Fiji	Fiji	2017	general population	Yes	https://www.sciencedirect.com/science/article/pii/S1201971219304345	Low chikungunya virus seroprevalence two years after emergence in Fiji	-
France	Martinique island and guadeloupe island	2013-2015	blood donors	Yes	https://journals.plos.org/plosntds/article?id=10.1371/journal.pntd.0005254#sec018	Epidemiology of Chikungunya Virus Outbreaks in Guadeloupe and Martinique, 2014	-
French Guyana	3 subregions across territory	2016	general population	Yes	https://www.nature.com/articles/s41467-020-16516-x	Reconstructing Mayaro virus circulation in French Guiana shows frequent spillovers	-
Gabon	Lamberéné	2015	general population	Yes	https://academic.oup.com/jid/article/227/2/261/6609553	Seroepidemiological Reconstruction of Long-term Chikungunya Virus Circulation in Burkina Faso and Gabon	-
Haiti	Ça Ira	2014	Healthy children cohort	No	https://www.ncbi.nlm.nih.gov/pmc/articles/PMC5096354/	Measuring Haitian children's exposure to chikungunya, dengue and malaria	Mathieu J. P. Poirier
Haiti	Throughout	2014-15	general population	No	https://www.ncbi.nlm.nih.gov/pmc/articles/PMC6004842/	Use of Bead-Based Serologic Assay to Evaluate Chikungunya Virus Epidemic, Haiti	-
India	Throughout	2017	general population	Yes	https://www.sciencedirect.com/science/article/pii/S2666524720301750	Seroprevalence of chikungunya virus infection in India, 2017: a cross-sectional population-based serosurvey	-
Kenya	Three districts	1966-68	general population	Yes	https://www.ncbi.nlm.nih.gov/pmc/articles/PMC2427766/pdf/bullwho00208-0028.pdf	A Multipurpose Serological Survey in Kenya	-
Kenya	3,200 Km2 semi-circle centered in the town of Busia	2/12/2010	general population	Yes	https://journals.plos.org/plosntds/article?id=10.1371/journal.pntd.0005998#sec002	Serological and spatial analysis of alphavirus and flavivirus prevalence and risk factors in a rural community in western Kenya	-
Kenya	Two village clusters	2009	general population	Yes	https://journals.plos.org/plosntds/article?id=10.1371/journal.pntd.0003436	High Rates of O'Nyong Nyong and Chikungunya Virus Transmission in Coastal Kenya	-
Kenya	Three districts	2004	general population	Yes	https://www.ncbi.nlm.nih.gov/pmc/articles/PMC3161961/	Seroprevalence and distribution of arboviral infections among rural Kenyan adults: A cross-sectional study	-
Malaysia	Ipoh (2006), Bagan Panchor (2006), Port Klang (1999) and Johor Baharu (2008)	study done in 2008	general population	Yes	https://link.springer.com/content/pdf/10.1186/1471-2334-13-67.pdf	Emergence of chikungunya seropositivity in healthy Malaysian adults residing in outbreak-free locations: Chikungunya seroprevalence results from the Malaysian Cohort	-

Mali	7 localities	2016	general population	No	https://www.nature.com/articles/s41467-021-26707-9	Model-based assessment of Chikungunya and O'nyong-nyong virus circulation in Mali in a serological cross-reactivity context	Natahanael Hozé
Nigeria	Ilorin	2019	Three hospitals	No	https://www.ajol.info/index.php/nvj/article/view/191737	Screening of immunoglobulin g antibodies against chikungunya virus among urban population in Ilorin Nigeria	Udeze Ao
Nigeria	Kainji Lake	1980	general population	Yes	https://academic.oup.com/trstmh/article-abstract/77/2/149/1915725?redirectedFrom=fulltext	Arthropod-borne virus antibodies in sera of residents of Kainji Lake Basin, Nigeria 1980	-
Philippines	Cebu	2010	general population	No	https://academic.oup.com/jid/article/213/4/604/2459450?login=false#google_vignette	Reconstruction of 60 Years of Chikungunya Epidemiology in the Philippines Demonstrates Episodic and Focal Transmission	-
Qatar	Throughout	2013-16	Blood donors	No	Dengue and chikungunya seroprevalence among Qatari nationals and immigrants residing in Qatar	Dengue and chikungunya seroprevalence among Qatari nationals and immigrants residing in Qatar	Gheyath Nasrallah
Rwanda	Throughout	2015	general population	No	https://www.liebertpub.com/doi/full/10.1089/vbz.2018.2393	Seroreactivity to Chikungunya and West Nile Viruses in Rwandan Blood Donors	Eric Seruyange
Senegal	Kedougou	2012	general population	Yes	https://www.mdpi.com/1999-4915/12/2/196/htm	Changes in the Transmission Dynamic of Chikungunya Virus in Southeastern Senegal	-
Senegal	Nomadic communities	2014	general population	Yes	https://www.mdpi.com/2076-0817/8/3/113/htm	Serological Data Shows Low Levels of Chikungunya Exposure in Senegalese Nomadic Pastoralists	-
Singapore	Singapore	2010	general population	Yes	Seroprevalence of antibodies against chikungunya virus in Singapore resident adult population (plos.org)	Seroprevalence of antibodies against chikungunya virus in Singapore resident adult population	-
Tanzania	Magugu in Babati rural in Manyara region and Wami-Dakawa in Mvomero district in Morogoro region	2019	general population	No	https://journals.plos.org/plosntds/article?id=10.1371/journal.pntd.0008061#sec002	Serological evidence of exposure to Rift Valley, Dengue and Chikungunya Viruses among agropastoral communities in Manyara and Morogoro regions in Tanzania: A community survey	Jaffu Chilongola
Tanzania	Mbeya region	2006-2011	general population	No	https://journals.plos.org/plosntds/article?id=10.1371/journal.pntd.0002979#s2	Seroprevalence of Alphavirus Antibodies in a Cross-Sectional Study in Southwestern Tanzania Suggests Endemic Circulation of Chikungunya	-

Tanzania	8 different areas	2018	general population	No	https://www.sciencedirect.com/science/article/pii/S1201971221006743	Seroprevalence and associated risk factors of chikungunya, dengue, and Zika in eight districts in Tanzania	Leonard Mboera
Vietnam	An Giang province, Ho Chi Minh City, Dak Lak province & Hue City	2015	Hospital patients	Yes	https://journals.plos.org/plosntds/article?id=10.1371/journal.pntd.0006246	Evidence of previous but not current transmission of chikungunya virus in southern and central Vietnam: Results from a systematic review and a seroprevalence study in four locations	-
Zambia	Lukanga swamps	2016	Vaccination campaign	Yes	https://journals.plos.org/plosone/article?id=10.1371/journal.pone.0235322	Sero-prevalence of arthropod-borne viral infections among Lukanga swamp residents in Zambia	-
Zambia	Trhoughout	2019	general population	No	unpublished		Samuel Bosomprah
