# Supplementary material for: Global burden of chikungunya virus infections and the potential benefit of vaccination campaigns
Source: Nat Med. 2025 Jun 10;31(7):2342–9. doi: 10.1038/s41591-025-03703-w (PMC12283390; doi:10.1038/s41591-025-03703-w)
Supplement: Supplementary file 4 — Supplementary Table 3 [file 41591_2025_3703_MOESM4_ESM.pdf]

A - Burden estimates per WHO Region						
**WHO Region**	**Classification**	**Infections**	**Cases**	**Deaths**	**DALYs**	**Chronic cases**
Africa	Endemic	1,860,000 (95% CI : 607,000-3,860,000)	931,000 (95% CI : 304,000-1,930,000)	106 (95% CI : 35-221)	15,800 (95% CI : 5,140-32,900)	44,700 (95% CI : 14,600-92,600)
Africa	Epidemic	6,450,000 (95% CI : 2,550,000-11,900,000)	3,220,000 (95% CI : 1,280,000-5,960,000)	381 (95% CI : 149-709)	53,600 (95% CI : 21,200-99,000)	155,000 (95% CI : 61,200-286,000)
Americas	Endemic	2,810,000 (95% CI : 216,000-8,560,000)	1,410,000 (95% CI : 108,000-4,280,000)	437 (95% CI : 34-1,330)	22,300 (95% CI : 1,710-67,900)	67,500 (95% CI : 5,180-205,000)
Americas	Epidemic	2,310,000 (95% CI : 421,000-5,240,000)	1,160,000 (95% CI : 210,000-2,620,000)	332 (95% CI : 58-770)	18,500 (95% CI : 3,350-41,900)	55,500 (95% CI : 10,100-126,000)
Eastern Mediterra-nean	Endemic	-	-	-	-	-
Eastern Mediterra-nean	Epidemic	4,140,000 (95% CI : 721,000-9,990,000)	2,070,000 (95% CI : 360,000-4,990,000)	322 (95% CI : 54-787)	34,200 (95% CI : 5,910-82,400)	99,300 (95% CI : 17,300-240,000)
Europe	Endemic	-	-	-	-	-
Europe	Epidemic	101.000 (95% CI : 0-324.00)	51.000 (95% CI : 0-162.00)	0.000 (95% CI : 0-0.00)	0.961 (95% CI : 0-3.08)	2.000 (95% CI : 0-8.00)

Southeast Asia	Endemic	9,090,000 (95% CI : 652,000-28,100,000)	4,550,000 (95% CI : 326,000-14,000,000)	1,040 (95% CI : 74-3,200)	70,600 (95% CI : 5,060-218,000)	218,000 (95% CI : 15,600-674,000)
Southeast Asia	Epidemic	5,900,000 (95% CI : 1,620,000-12,800,000)	2,950,000 (95% CI : 811,000-6,400,000)	747 (95% CI : 196-1,630)	46,600 (95% CI : 12,900-101,000)	142,000 (95% CI : 38,900-307,000)
Western Pacific	Endemic	-	-	-	-	-
Western Pacific	Epidemic	2,770,000 (95% CI : 312,000-6,940,000)	1,390,000 (95% CI : 156,000-3,470,000)	330 (95% CI : 33-845)	22,800 (95% CI : 2,560-57,000)	66,600 (95% CI : 7,500-167,000)
Global	Endemic	13,800,000 (95% CI : 3,830,000-33,100,000)	6,880,000 (95% CI : 1,920,000-16,500,000)	1,580 (95% CI : 386-3,820)	109,000 (95% CI : 30,900-259,000)	330,000 (95% CI : 92,000-793,000)
Global	Epidemic	21,600,000 (95% CI : 12,900,000-32,400,000)	10,800,000 (95% CI : 6,450,000-16,200,000)	2,110 (95% CI : 1,210-3,280)	176,000 (95% CI : 105,000-262,000)	518,000 (95% CI : 310,000-776,000)
**Global**	**Endemic and epidemic**	**35,300,000 (95% CI : 20,900,000-56,500,000)**	**17,700,000 (95% CI : 10,500,000-28,200,000)**	**3,690 (95% CI : 2,060-6,110)**	**284,000 (95% CI : 170,000-451,000)**	**848,000 (95% CI : 502,000-1,360,000)**

B - Vaccine impact per WHO Region

WHO region	classific-ation	Doses required	Infections averted	Cases averted	Deaths averted	DALYs averted	Chronic cases averted
Africa	Endemic	6,620,000.0 (95% CI : 6,620,000.0-6,620,000.0)	286,000.0 (95% CI : 179,000.0-351,000.0)	167,000.0 (95% CI : 118,000.0-198,000.0)	22.8 (95% CI : 13.5-28.3)	2,580.0 (95% CI : 1,630.0-3,150.0)	8,040.0 (95% CI : 5,670.0-9,500.0)
Africa	Epidemic	26,800,000.0 (95% CI : 23,700,000.0-29,900,000.0)	1,700,000.0 (95% CI : 1,520,000.0-1,880,000.0)	975,000.0 (95% CI : 874,000.0-1,070,000.0)	133.0 (95% CI : 119.0-146.0)	14,200.0 (95% CI : 12,700.0-15,600.0)	46,800.0 (95% CI : 41,900.0-51,400.0)
Americas	Endemic	14,200,000.0 (95% CI : 14,200,000.00-14,200,000.0)	326,000.0 (95% CI : 82,000.00-409,000.0)	227,000.0 (95% CI : 101,000.00-256,000.0)	25.0 (95% CI : 6.47-35.6)	2,800.0 (95% CI : 773.00-3,440.0)	10,900.0 (95% CI : 4,840.00-12,300.0)
Americas	Epidemic	10,100,000.0 (95% CI : 8,030,000.00-12,400,000.0)	465,000.0 (95% CI : 345,000.00-575,000.0)	274,000.0 (95% CI : 208,000.00-337,000.0)	39.1 (95% CI : 30.30-47.6)	3,720.0 (95% CI : 2,760.00-4,610.0)	13,100.0 (95% CI : 10,000.00-16,200.0)
Eastern Mediterranean	Endemic	-	-	-	-	-	-
Eastern Mediterranean	Epidemic	12,700,000 (95% CI : 10,500,000.0-15,200,000.0)	736,000 (95% CI : 594,000.0-862,000.0)	425,000 (95% CI : 346,000.0-496,000.0)	57 (95% CI : 46.7-66.2)	6,100 (95% CI : 4,930.0-7,160.0)	20,400 (95% CI : 16,600.0-23,800.0)
Europe	Endemic	-	-	-	-	-	-

Europe	Epidemic	0 (95% CI : 0-0)	0 (95% CI : 0-0)	0 (95% CI : 0-0)	0 (95% CI : 0-0)	0 (95% CI : 0-0)	0 (95% CI : 0-0)
Southeast Asia	Endemic	33,100,000.0 (95% CI : 33,100,000.0-33,100,000.0)	987,000.0 (95% CI : 282,000.0-1,190,000.0)	638,000.0 (95% CI : 303,000.0-718,000.0)	73.1 (95% CI : 20.2-95.6)	8,290.0 (95% CI : 2,590.0-9,870.0)	30,600.0 (95% CI : 14,500.0-34,500.0)
Southeast Asia	Epidemic	19,400,000.0 (95% CI : 16,600,000.0-22,700,000.0)	914,000.0 (95% CI : 741,000.0-1,080,000.0)	537,000.0 (95% CI : 444,000.0-631,000.0)	70.7 (95% CI : 58.4-83.6)	7,230.0 (95% CI : 5,880.0-8,580.0)	25,800.0 (95% CI : 21,300.0-30,300.0)
Western Pacific	Endemic	-	-	-	-	-	-
Western Pacific	Epidemic	8,930,000.0 (95% CI : 6,900,000.0-11,000,000.0)	437,000.0 (95% CI : 310,000.0-554,000.0)	257,000.0 (95% CI : 193,000.0-324,000.0)	33.9 (95% CI : 25.6-42.6)	3,590.0 (95% CI : 2,560.0-4,550.0)	12,300.0 (95% CI : 9,280.0-15,500.0)
**Global**	Endemic	53,900,000 (95% CI : 53,900,000.0-53,900,000)	1,600,000 (95% CI : 870,000.0-1,860,000)	1,030,000 (95% CI : 668,000.0-1,140,000)	121 (95% CI : 65.6-148)	13,700 (95% CI : 7,550.0-15,700)	49,600 (95% CI : 32,100.0-54,700)
**Global**	Epidemic	78,000,000 (95% CI : 71,900,000.0-84,100,000)	4,250,000 (95% CI : 3,910,000.0-4,570,000)	2,470,000 (95% CI : 2,290,000.0-2,640,000)	333 (95% CI : 310.0-356)	34,800 (95% CI : 32,000.0-37,300)	118,000 (95% CI : 110,000.0-127,000)
**Global**	**Endemic and epidemic**	**132,000,000 (95% CI : 126,000,000.0-138,000,000)**	**5,850,000 (95% CI : 5,080,000.0-6,310,000)**	**3,500,000 (95% CI : 3,120,000.0-3,730,000)**	**454 (95% CI : 394.0-493)**	**48,500 (95% CI : 42,100.0-52,000**)	**168,000 (95% CI : 150,000.0-179,000)**

C - Vaccine impact per dose used					
**Classification**	**Infections averted per 100,000 doses**	**Cases averted per 100,000 doses**	**Deaths averted per 100,000 doses**	**DALYs averted per 100,000 doses**	**Chronic cases averted per 100,000 doses**
Endemic	2,970 (95% CI : 1,610-3,440)	1,920 (95% CI : 1,240-2,110)	0.224 (95% CI : 0.122-0.274)	25.4 (95% CI : 14.0-29.1)	92 (95% CI : 59.5-101)
Epidemic	5,460 (95% CI : 5,010-5,980)	3,170 (95% CI : 2,910-3,450)	0.428 (95% CI : 0.395-0.463)	44.7 (95% CI : 41.0-48.7)	152 (95% CI : 140.0-165)
**Endemic and Epidemic**	**4,440 (95% CI : 3,820-4,800)**	**2,650 (95% CI : 2,350-2,830)**	**0.345 (95% CI : 0.298-0.373)**	**36.8 (95% CI : 31.8-39.7)**	**127 (95% CI : 113.0-136)**
