# Supplementary material for: Author Correction: Global burden of chikungunya virus infections and the potential benefit of vaccination campaigns
Source: Nat Med. 2025 Nov 10;31(12):4312. doi: 10.1038/s41591-025-04065-z (PMC12705449; doi:10.1038/s41591-025-04065-z)
Supplement: Supplementary file 1 — Original, uncorrected Figs. 3–5 and Extended Data Fig. 3 [file 41591_2025_4065_MOESM1_ESM.pdf]

nature medicine

Supplementary information

Author Correction: Global burden of chikungunya virus infections and the potential benefit of vaccination campaigns

In the format provided by the authors and unedited

Original, uncorrected figures

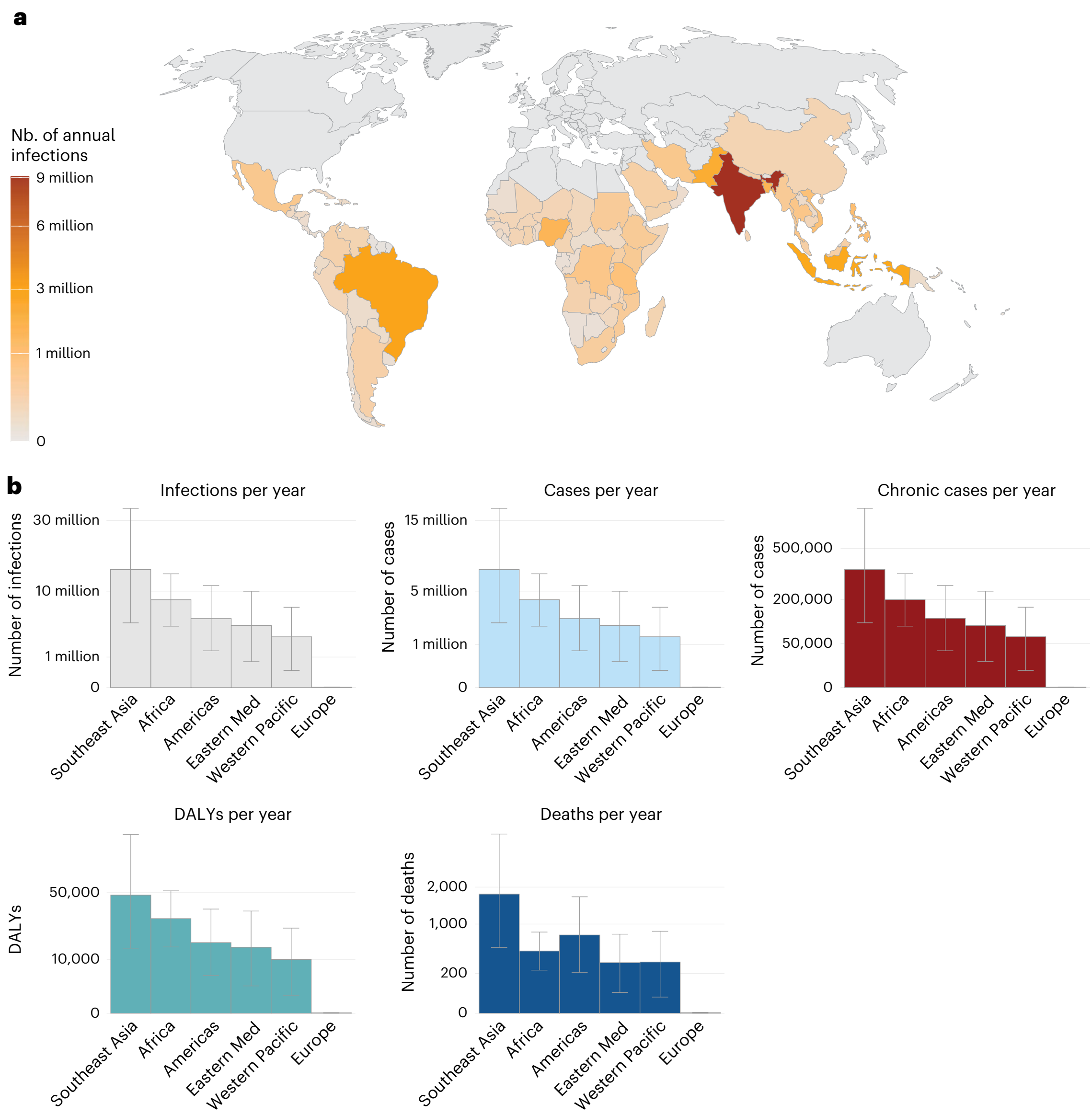

Fig. 3 | Burden estimates.

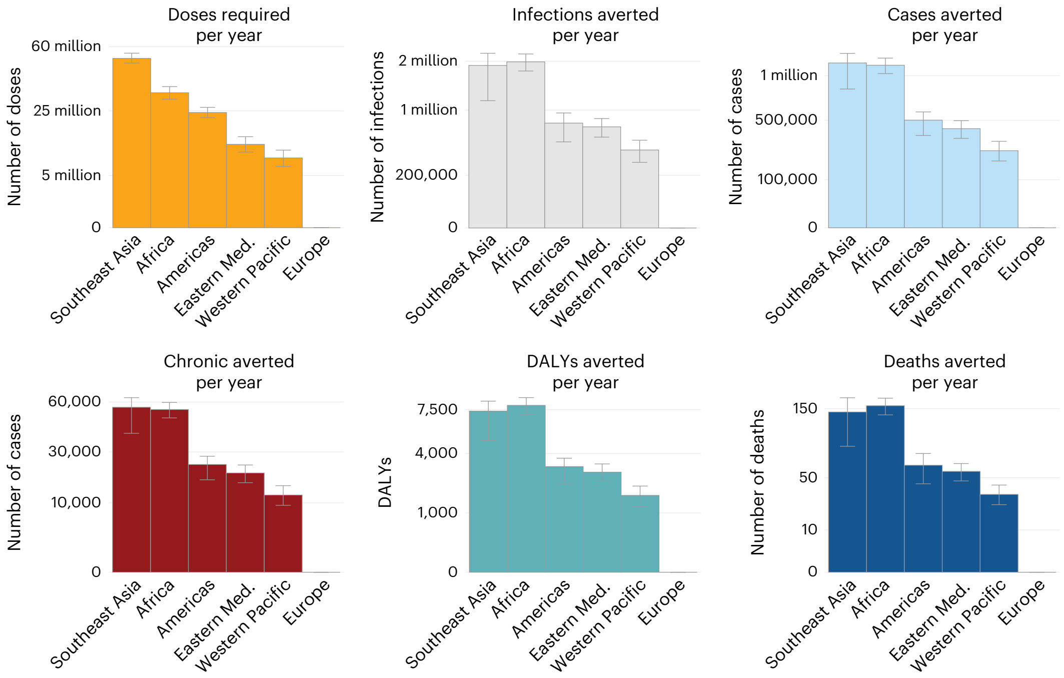

Fig. 4 | Summary of impact by WHO region for the base case model.

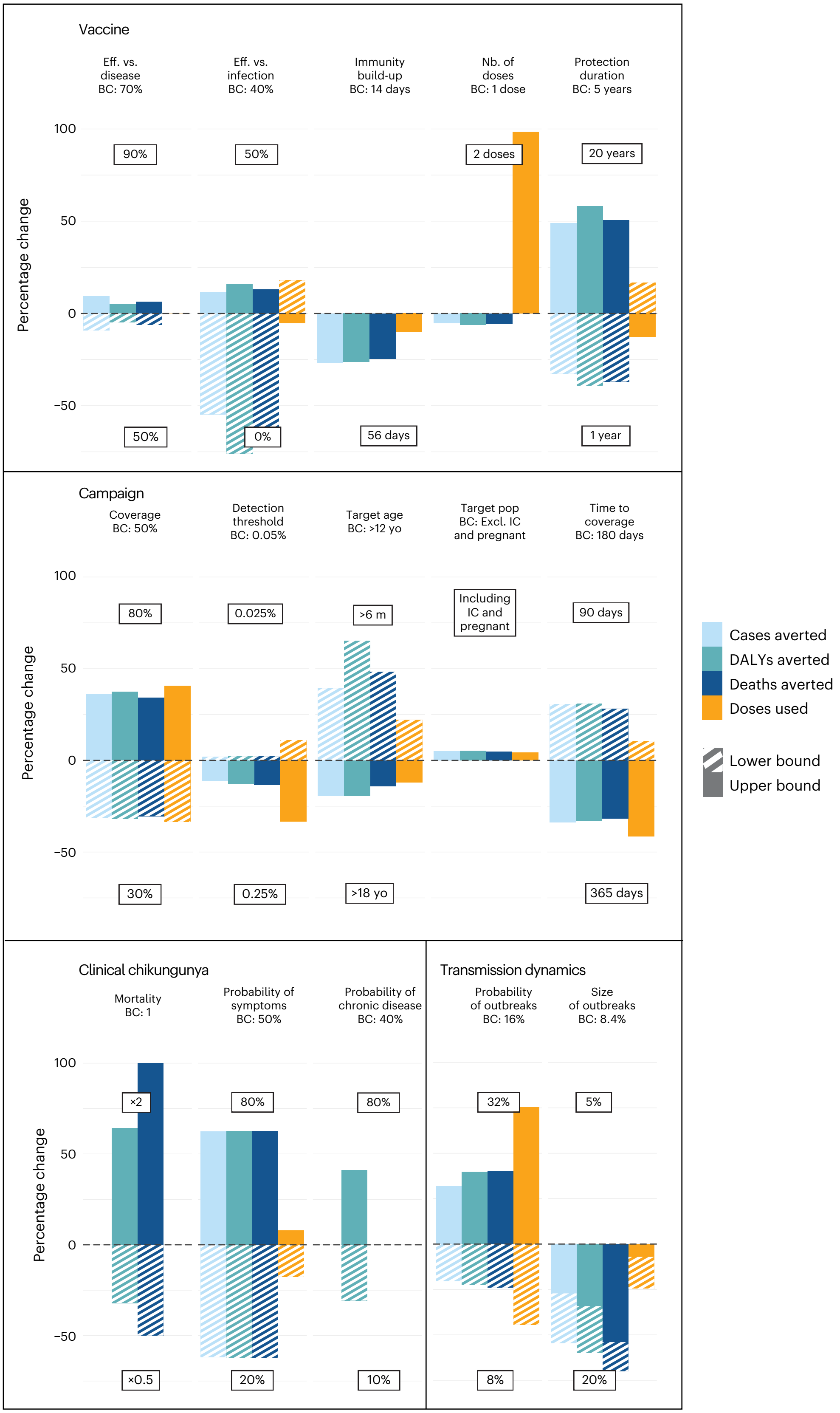

Fig. 5 | Sensitivity analysis of the model parameters.

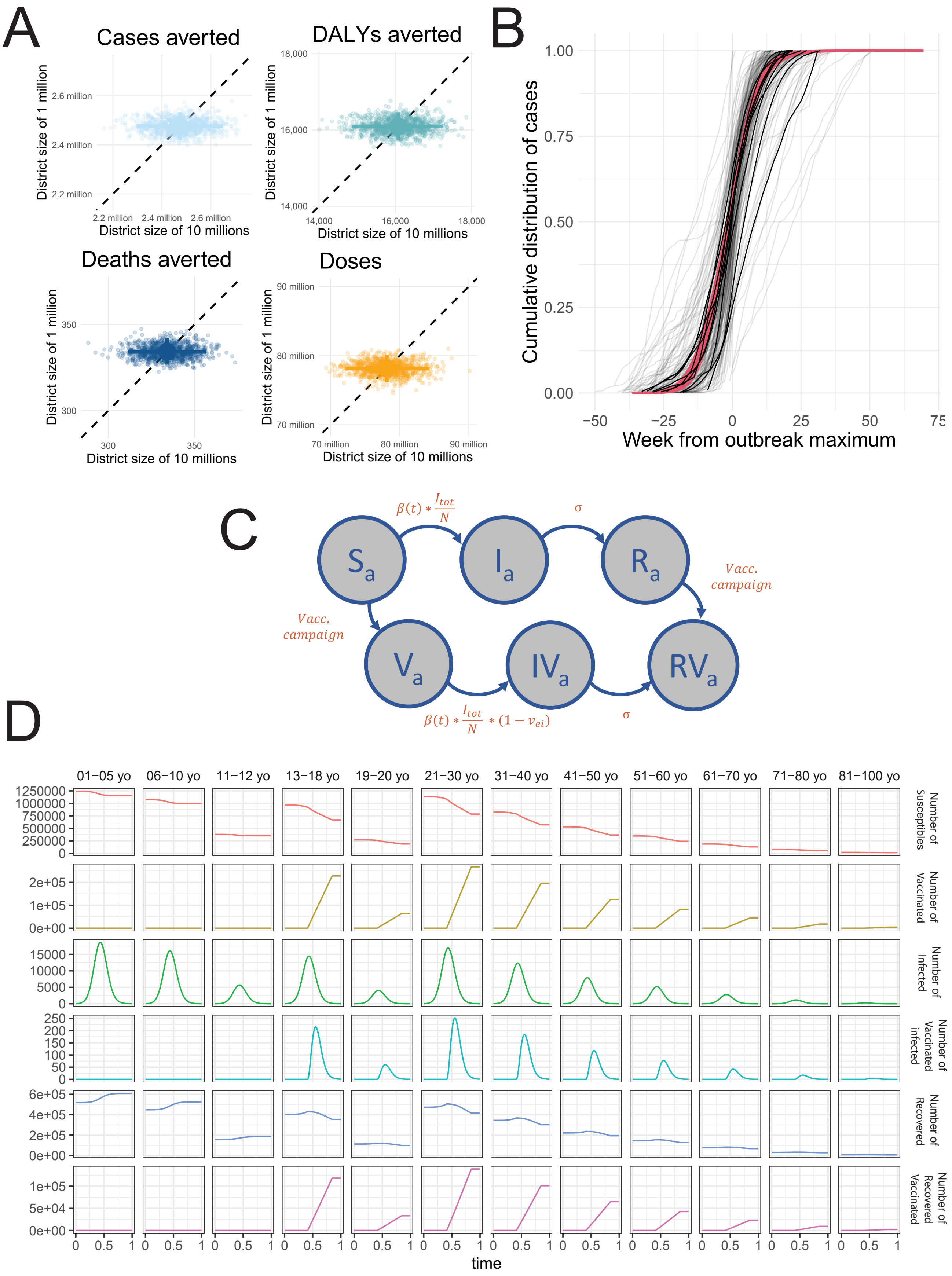

Extended Data Fig. 3 | Details on the simulation framework.
